# Phage T7 DNA mimic protein Ocr is a potent inhibitor of BREX defence

**DOI:** 10.1093/nar/gkaa290

**Published:** 2020-04-27

**Authors:** Artem Isaev, Alena Drobiazko, Nicolas Sierro, Julia Gordeeva, Ido Yosef, Udi Qimron, Nikolai V Ivanov, Konstantin Severinov

**Affiliations:** 1 Skolkovo Institute of Science and Technology, Moscow 143028, Russia; 2 Philip Morris International R&D, Philip Morris Products S.A., Neuchatel 2000, Switzerland; 3 Department of Clinical Microbiology and Immunology, Sackler Faculty of Medicine, Tel Aviv University, Tel Aviv 69978, Israel; 4 Waksman Institute of Microbiology, Piscataway, NJ 08854, USA; 5 Institute of Gene Biology, Russian Academy of Sciences, Center for Precision Genome Editing and Genetic Technologies for Biomedicine, Institute of Gene Biology, Russian Academy of Sciences, 34/5 Vavilov str., 119334 Moscow, Russia

## Abstract

BREX (for BacteRiophage EXclusion) is a superfamily of common bacterial and archaeal defence systems active against diverse bacteriophages. While the mechanism of BREX defence is currently unknown, self versus non-self differentiation requires methylation of specific asymmetric sites in host DNA by BrxX (PglX) methyltransferase. Here, we report that T7 bacteriophage Ocr, a DNA mimic protein that protects the phage from the defensive action of type I restriction–modification systems, is also active against BREX. In contrast to the wild–type phage, which is resistant to BREX defence, T7 lacking Ocr is strongly inhibited by BREX, and its ability to overcome the defence could be complemented by Ocr provided *in trans*. We further show that Ocr physically associates with BrxX methyltransferase. Although BREX+ cells overproducing Ocr have partially methylated BREX sites, their viability is unaffected. The result suggests that, similar to its action against type I R–M systems, Ocr associates with as yet unidentified BREX system complexes containing BrxX and neutralizes their ability to both methylate and exclude incoming phage DNA.

## INTRODUCTION

BREX (BacteRiophage EXclusion) is a poorly studied bacterial and archaeal defence system. A prototypic system of this kind, referred to at the time as Pgl (Phage Growth Limitation), was discovered in *Streptomyces coelicolor A3(2)* and postulated to act in a way that is reciprocal to the defence provided by restriction–modification (R–M) systems (1,2). R–M systems protect cells by epigenetic modification (methylation) of specific sites in the genome. Incoming phage DNA lacking these modifications is subject to restriction—cleavage by endonucleases that do not recognize the modified DNA of the host (3). The Pgl system is believed to first modify the incoming phage DNA and then inhibit, through an unknown mechanism, the appearance of progeny of the re-infecting modified phage (4).

Bioinformatics searches in the neighborhoods of genes encoding PglZ—a core component of Pgl—have uncovered multiple putative phage defence systems, united under a common name BREX (5,6). Two of these systems, from *Bacillus cereus* and *Escherichia coli*, were shown to protect their hosts from infection by diverse phages (6,7). Each was shown to methylate a specific (and distinct) asymmetric site in host DNA. In the case of the *E. coli* system, methylation of phage DNA at BREX sites was shown to be sufficient for overcoming BREX defence (7). Both BREX systems prevent the appearance of replicated phage DNA, although whether this occurs through destruction of incoming unmodified phage genome or inhibition of its replication is not known.

**Figure 1.**
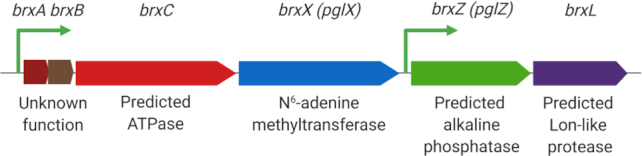
Structure of a type I BREX gene cluster from *Escherichia coli* HS. Operon structure with predicted promoter positions is shown. Genes *brxA* and *brxB* are translationally coupled.

Evolutionarily, the variety of BREX systems can be reduced to six classes differing in the sets of proteins they encode. The minimal set of genes required for type I BREX protection includes BrxX (PglX), a SAM-dependent methyltransferase; BrxZ (PglZ), a putative alkaline phosphatase; BrxC, a putative ATPase; BrxL, a putative Lon-like protease; and two small proteins with unknown function (Figure 1). BREX systems of five classes encode the BrxX methyltransferase, indicating that DNA modification might be a common way for these systems to distinguish self from non-self, which makes them, in this respect, similar to R–M systems. However, none of the BREX system proteins resemble known nucleases that are responsible for the defensive functions of R–M systems, indicating that BREX might provide protection from (exclude) phages via a novel mechanism(s) that remains to be established.

Whatever the defence mechanism of BREX, it must be activated by the presence of phage DNA containing unmodified BREX sites. These sites must be recognized by the BrxX methyltransferase, alone or in complex with other proteins, leading to either BREX site methylation or exclusion of the incoming DNA. Because BREX sites are asymmetrical and methylated at only one DNA strand, half of the sites will become unmethylated after replication and must be remethylated to prevent damage to the host. The type I and type III R–M systems also recognize and methylate asymmetric sites in DNA (8–10). The type I methylation complex consists of subunits HsdS and HsdM in a 1 to 2 stoichiometry and is capable of methylation of non-palindromic cognate recognition sequence at both DNA strands. When bound to two HsdR subunits, the HsdM_2_HsdS_1_ complex recognizes unmodified sites, and through the function of ATP-dependent HsdR motor pulls on the DNA creating a loop (11). When two HsdM_2_HsdS_1_HsdR_2_ complexes pulling from different sites collide or a roadblock is encountered by one complex pulling on the DNA, cleavage around the site of collision occurs (12). Post-replication cleavage of host DNA is avoided since the HsdM_2_HsdS_1_ and HsdM_2_HsdS_1_HsdR_2_ complexes have a strong preference for methylating hemi-methylated sites over fully unmethylated sites, promoting the maintainance of host modification over modification of incoming foreign DNA. The type III R–M systems function similarly but methylation of their recognition sites occurs only on one DNA strand, which is similar to situation during self versus non-self distinction by BREX. To ensure that the host DNA is not attacked after replication the type III R–M complexes rely on specific relative orientation of individual recognition sites. Only the interaction between two restriction complexes bound to sites in an inverted-repeat orientation (head-to-head or tail-to-tail) results in efficient cleavage at the collision site (13). Whether specific site orientation is required for BREX exclusion is not known. An unrelated example of an R–M system that modifies only one strand of DNA is Type IIL, where restriction endonuclease and methyltransferase activities are combined in one polypeptide (14,15). How these systems manage to avoid post-replicational self cleavage is not clear.

Phages have evolved multiple strategies to counter host defences (16,17). In the case of type I R–M systems, phage-encoded proteins that mimic DNA bind the restriction complex and prevent its binding to unmodified recognition sites in phage DNA (18–20). DNA binding and methylation by the methylation complex is also prevented. A prototypical protein of this kind is Ocr (‘overcome classical restriction’), the product of the lytic bacteriophage T7 gene *0.3*. This 117-amino acid protein forms a dimer with each monomer mimicking in shape and surface charge distribution one helical turn of double-stranded DNA (21,22). The dimer thus corresponds to ∼20 bp of DNA, whose trajectory is slightly bent at the interface between the monomers. Being a DNA mimic, Ocr binds many host proteins that interact with nucleic acids (e.g. DNA-dependent RNA polymerase (23)) and might thus be an ideal multipurpose inhibitor of cellular defence systems that depend on recognition of viral DNA for their function. Here, we show that Ocr, but not several other DNA mimic antirestriction proteins, overcomes *E. coli* BREX defence by specifically binding BrxX methyltransferase. In the presence of Ocr, cells carrying a BREX system contain partially modified DNA, and, yet, their viability is not affected. Phages induced from such cells are sensitive to BREX defence, though the level of protection is decreased relative to that seen with an unmodified phage. The results suggest that, similar to type I R–M systems, BREX action relies on the kinetic interplay of at least two types of complexes, one able to methylate DNA and another that can act against both unmodified and partially modified DNA. Additionally, similar to type I R–M systems, Ocr must be inhibiting both types of BREX complexes.

## MATERIALS AND METHODS

### Bacterial strains, plasmids and phages

See Supplementary Table S1 for a full list of bacterial strains, plasmids and phages used in this study. *E. coli* BW25113 (EcoKI R– M+) and AB1157 (EcoKI R+ M+) were used throughout. *Escherichia coli* XL1 Blue chemically competent cells were obtained from Evrogen (Russia). *Escherichia coli* NEB5α and T7 express strains were obtained from New England Biolabs (USA). Unless otherwise indicated, cells were grown at 37°C in standard LB media with appropriate antibiotics. The pBREX AL plasmid was described previously (7), it is based on a low copy number vector pBTB-2 and carries the entire *E. coli* HS BREX cluster with its native promoters. BW25113 transformed with pBREX AL is referred to as BREX+. BW25113 transformed with the pBTB-2 vector is referred to as BREX–. Plasmids pArdA, pArdB, pArn and pOcr F54D/A58E were a kind gift from Dr Gennady Zavilgelsky (State Research Institute for Genetics and Selection of Industrial Microorganisms, Moscow, Russia). An EcoKI–*E. coli* strain T7 express was used to obtain λ phage without BREX- or EcoKI-specific modifications. Other phages, including T7, were propagated on the BW25113 strain.

### Plasmid construction

See Supplementary Table S2 for a list of primers used in this study. Plasmid pUC-0.3 was constructed to delete gene *0.3* in the T7 genome by replacing it with the *trxA* gene, a positive selection marker for T7 growth on hosts lacking *trxA*. The *trxA* in pUC-0.3 is flanked by 50-bp sequences upstream and downstream of gene *0.3* in its natural context in phage genome. The plasmid was constructed by Gibson Assembly with linearized pUC-19 backbone and PCR product of the *trxA* gene from T7 genome amplified by using primers IY27F and IY27R. Plasmids for overproduction of wild**-**type Ocr, wild-type Ocr with C-terminal Strep-tag II (WSHPQFEK), or Ocr F54D/A58E mutant were constructed using NEBuilder HiFi DNA Assembly Master Mix (New England Biolabs, USA). All versions of *0.3* gene were cloned under control of the araBAD promoter into the pBAD L24 vector—a modified version of pBAD/HisB (Invitrogen, USA). The *0.3* gene was amplified from T7 genome by using primers Ocr_F and Ocr_R or Ocr_R_Strep. Ocr F54D/A58E was amplified from the pOcr F54D/A58E by using Ocr_F and Ocr_R primers. PCR-products were purified with GeneJET PCR Purification Kit (K0702, Thermo Scientific, USA) and assembled with EcoRI linearized pBAD L24 backbone. Strep-tag II was added to C-termini of BrxB, BrxZ, BrxL or BrxX in the context of the entire BREX cluster in pBREX AL plasmid. In each case, two PCR products with a ∼20-bp overlap were amplified from pBREX AL vector at the first stage, with one of them carrying a Strep-tag coding sequence. In the second stage, purified products were used for overlap-extension PCR: for the first 6 cycles reaction was carried without primers at *T*_a_ = 60°C, followed by 20 cycles at *T*_a_ = 72°C, after addition of the flanking primers. Resulting insert was cloned into the pBREX AL by endonuclease restriction digestion and ligation. To create pBREX AL BrxB C-Strep, primers 5′ Sac I + BrxB_R_Strep and BrxB_F + 3′ Xba I were used in the first stage and primers 5′ Sac I + 3′ Xba I in the second stage; the insert was introduced between the Sac I and Xba I sites of pBREX AL. To create pBREX AL BrxX C-Strep, primers 5′ Xba I + BrxX_R_Strep and BrxX_F + 3′ Nhe I were used in the first stage and 5′ Xba I + 3′ Nhe I in the second stage; the insert was introduced between the Xba I and Nhe I sites of pBREX AL. To create pBREX AL BrxZ C-Strep, primers 5′ Nhe I + BrxZ_R_Strep and BrxZ_F + 3′ Bgl II were used in the first stage and 5′ Nhe I + 3′ Bgl II in the second stage; the insert was introduced between the Nhe I and Bgl II sites of pBREX AL. To create pBREX AL BrxL C-Strep, primers 5′ Not I + BrxL_R_Strep and BrxL_F + 3′ Sac I were used in the first stage and 5′ Not I + 3′ Sac I in the second stage; the insert was introduced between the Not I and Sac II sites of pBREX AL. XL1 Blue chemically competent *E. coli* cells were used for transformation followed by selection of desired clones. All final plasmids were confirmed by Sanger sequencing.

### Construction of bacteriophage T7 lacking gene *0.3*

A T7 bacteriophage lacking gene *0.3* was constructed by homologous recombination. Plasmid pUC-0.3 was transformed into *E. coli* strain NEB5α. Cells were grown overnight in LB medium supplemented with 100 μg/ml ampicillin at 37°C. The culture was diluted 1:20 in fresh LB medium supplemented with 100 μg/ml ampicillin and growth was continued at 42°C until OD_600_ reached 0.5. Cells were infected with wild-type phage T7 at a multiplicity of infection (MOI) of 0.1. The cultures remained at 42°C until complete lysis. The resulting lysate was used to infect BW25113 *E. coli* lacking *trxA* (BW25113Δ*trxA* from KEIO collection) to select phages that had recombined the fragment containing *trxA* into their genome in place of gene *0.3*. Before infection, BW25113Δ*trxA* was grown overnight in LB supplemented with 25 μg/ml kanamycin at 37°C. The overnight culture was diluted 1:1 in 3 ml of fresh LB medium supplemented with 25 μg/ml kanamycin at 37°C and allowed to grow for 1 hour. The culture was then centrifuged at ∼4500 × g at 4°C for 10 min. Cell pellet was resuspended in 3 ml of LB supplemented with 0.7% (w/v) agar. 0.5 mL of T7 lysate, containing recombinant phage (prepared as described above), was added to the suspension. Since thioredoxin, encoded by *trxA*, is an essential co-factor of the T7 DNA-Polymerase, this procedure selected for phages that had recombined the fragment containing *trxA* into their genome in place of gene *0.3*. The suspension was poured onto an LB agar plate and incubated at 37°C for 3 h. Single plaques from several emerging on the plate were purified, and the presence of desired insertion was verified by DNA sequencing.

### Generation of secondary mutants of the T7 *0.3-0.7* fusion phage

Secondary mutants with increased efficiency of BREX+ culture lysis were selected in the course of multiple re-infections with parental T7 *0.3–0.7* fusion line from laboratory stock. 10 ml of fifteen independent BREX+ cultures were infected with 100 μl of T7 *0.3–0.7* fusion lysate (∼10^10^ pfu/ml) at OD_600_ = 0.6 (MOI ∼ 1) and grown overnight at 37°C. 100 μl of culture supernatants collected the next day were used to infect fresh BREX+ culture. Though lysis of BREX+ cultures was observed as early as after two rounds of infection, the experiment was continued for five rounds. Phages were purified from single plaques grown on a BREX+ lawn and propagated in a BREX– liquid culture to eliminate possible BREX system-specific modifications.

### Phage infection experiments

Overnight bacterial cultures were diluted 100-fold in LB medium with appropriate antibiotics and grown at 37°C. For λ_vir_ infection, the medium was supplemented with 0.2% maltose and 5 mM MgCl_2_. After OD_600_ reached 0.6, 200 μl aliquots were transferred to 96-well plates and infected at desired MOI. Optical density was monitored for 20 h using the EnSpire Multimode Plate Reader (PerkinElmer, USA). All infections were performed in three biological replicas. To study the effects of anti-restriction protein production on phage infection, cultures were initially grown without induction. After 1-hour growth in LB medium, cultures were supplemented with 1 mM IPTG or 13.3 mM l-arabinose (unless otherwise indicated), grown for additional 2 hours (reaching an OD_600_ ∼ 0.6), and then infected with the phage.

### Efficiency of plating (EOP) assay

To measure phage titers and estimate the efficiency of BREX or EcoKI system protection, EOP assay was performed. Overnight cultures (100 μl) were mixed with 10 ml of 0.6% top LB agar supplemented with appropriate antibiotics and poured on the surface of precast 1.2% bottom LB agar plates. 10 μl drops of serial 10-fold phage lysate dilutions were spotted on the top agar, allowed to dry and plates were incubated at 37°C overnight. All experiments were performed in three biological replicates. The level of protection was determined as the ratio of phage titers obtained on a non-restrictive (BREX–) host to that on restrictive (BREX+ or EcoKI+) hosts.

### Protein pull-down

Overnight cultures of *E. coli* strains with plasmids encoding the Strep-tagged protein and its candidate interaction partner (pBAD Ocr wt C-strep + pBREX AL and pBAD Ocr wt + pBREX AL B/X/Z/L–Strep) were diluted 100-fold in 500 ml of fresh LB medium supplemented with antibiotics and grown in 1 L flasks to OD_600_∼0.3. At this point, Ocr/Ocr C-strep expression was induced with 13.3 mM of l-arabinose. After induction, cultures were grown until OD_600_ ∼0.6. In an experiment that involved phage infection, wild-type T7 was added at MOI = 1 and infection was allowed to proceed for 15 min. Cells were harvested by centrifugation at 4000 × g at 4°C for 10 min, cell pellets were washed in 10 ml of buffer A (150 mM NaCl, 1 mM EDTA, 100 mM Tris–HCl; pH 8.0), and resuspended in the same buffer supplemented with a protease inhibitor cocktail (Roche). Cells were disrupted using Emulsiflex C5 (Avestin, Germany) homogenizer (5× cycles under 20 000 psi pressure), and the lysate was clarified by centrifugation at 15 000 × g at 4°C for 30 min. The supernatant was applied to an equilibrated 1-ml StrepTrap HP chromatography column, which was then washed with 20 column volumes of buffer A. Elution was performed by applying a gradient concentration of buffer B (150 mM NaCl, 1 mM EDTA, 100 mM Tris–HCl, 2.5 mM desthiobiotin; pH 8.0). Protein-containing fractions were concentrated using 10-kDa Amicon centrifugal filter units (Merk, USA) and analyzed by SDS-PAGE on a 4–20% gradient gel. The identity of protein bands was determined by matrix-assisted laser desorption/ionization time-of-flight (MALDI-TOF) mass spectrometry. Samples were prepared with Trypsin Gold (Promega, USA) in accordance with manufacturer's instructions. Mass spectra were obtained using the rapifleX system (Bruker, USA).

### Genomic DNA purification and sequencing

High-titer phage lysates (10 ml; ∼10^10^ pfu/ml) were obtained for purification of genomic DNA. Phage particles were precipitated with 10% polyethylene glycol 8000, 1 M NaCl and DNA was extracted with phenol:chloroform, as described elsewhere (24). DNA libraries were prepared in accordance with a standard protocol and sequenced by using MiniSeq platform (Illumina, USA) with paired-end 150 cycles (75 + 75). Genome assembly was performed using SPAdes (25). To detect mutations within the T7 genome, variant calling against the reference genome was performed using SAMtools and BCFtools (https://github.com/samtools).

### Pacific bioscience sequencing

Modified BREX and EcoKI motifs were determined using the PacBio platform. Bacterial cultures were grown in triplicates in 10 ml of LB medium supplemented with appropriate antibiotic at 37°C for 1 h. Ocr expression was induced for an additional 2 h with 13.3 mM l-arabinose. Total DNA was purified from 2 ml of cultures using the GeneJET Genomic DNA Purification Kit (K0722, Thermo Scientific, USA) in accordance with manufacturer's instructions. The extracted DNA was sheared to a targeted mean size of 500 bp (the effective subreads size was on average of 972 bp (min: 849, median: 956, max: 1240)) using ultrasonicator (Covaris, USA) and purified with AMPure PB beads (Pacific Biosciences, USA). PacBio sequencing libraries were prepared using the SMRTbell Template Prep kit 1.0 (Pacific Biosciences, USA). Protocols for polymerase binding were generated by the Pacific Biosciences Binding Calculator. Sequencing was performed using PacBio RS II (Pacific Biosciences, USA), each sample being sequenced for six hours on one SMRT cell. The PacBio SMRT Analysis software was used for alignment of reads and detection of base modification. The percentage of modified BREX, EcoKI and Dam motifs was determined using the coverage and modification QV reported in the ‘modifications.csv.gz’ file produced by the analysis software. For each motif, methylation-targeted adenines with a reported coverage lower than 25 were discarded, and the remaining adenines were considered as modified if their modification QV was at least 30. Sequencing statistics are available in the Supplementary Tables S3–S7.

### Verification of phage λ modification status

*Escherichia coli* BW25113 cells were lysogenized with temperature-sensitive λ phage cI857 *bor*::*Cm* (7). The lysogen was transformed with pBTB-2/pBREX AL and pBAD Ocr or pBAD Ocr F54D/A58E plasmids. The cultures were grown at 30°C to an OD_600_ 0.6 in 10 ml LB medium supplemented with appropriate antibiotics in the presence of indicated l-arabinose concentrations to induce Ocr expression. λ prophage induction was achieved by incubating cells at 42°C for 60 min, until lysis was observed, and cultures were then additionally treated with 100 μl of chloroform. The lysate was cleared by centrifugation, and phage titers were determined on lawns of BREX-, BREX+ or AB1157 cells on LB plates supplemented with 0.2% maltose and 5 mM MgCl_2_. The plates were incubated overnight at 37°C and the level of protection was estimated as the ratio of phage titers obtained on non-restrictive (BREX-) host relative to restrictive (BREX+ or AB1157) hosts.

## RESULTS

### Productive infection of BREX culture by phage T7 requires the presence of intact *0.3* gene

The *E. coli* BREX system provides protection against a broad range of double-stranded DNA phages with lytic and temperate life cycles. Known exceptions are phages T4 and VR5 with glycosylated DNA (7,26). In a previous communication, we reported that the *E. coli* BREX system protected cells from infection with what we at the time considered wild-type T7 phage (7). While studying the infection of BREX cells by several T7 isolates from our laboratory collection, we identified phages that fully overcame the BREX defence. We determined the genomes of phages from several BREX-sensitive stocks and of BREX-resistant variants. Surprisingly, we found the genomes of BREX-resistant phages to match the wild-type T7 sequence, while all sensitive phages harbored a deletion in the early region (positions 1256–2735). This deletion has been previously described in various publications and is thought to be associated with increased fitness (deletion H1 of Studier; 27–29). It arises spontaneously during laboratory cultivation in a rich medium at 37°C. In the wild-type phage, the deleted material is flanked by two 12-bp direct repeats (AGGAAGTCGAGG; Supplementary Figure S1A). Therefore, the deletion might have arisen because of DNA polymerase slippage during phage DNA replication, as repeats are too short to promote homologous recombination (30). Deletion leads to complete loss of genes *0.4*, *0.5*, and *0.6* and an in-frame fusion of genes *0.3* and *0.7*. The fused ORF encodes a 231 amino acid protein, consisting of 110 N-terminal amino acids of the 117 amino-acid Gp0.3 and 121 C-terminal amino acids of the 359 amino-acid Gp0.7. We will refer to T7 strain with this deletion as the ‘*0.3–0.7* fusion’ in this manuscript. The *0.3* gene encodes a DNA mimic anti-restriction protein, Ocr, which inactivates type I R–M enzymes (21). Gene *0.7* encodes a protein kinase, which phosphorylates the host RNA polymerase β’ subunit as well as other host and viral proteins and also contributes to a timely switch from host to viral RNA polymerase transcription (31,32). The part of the *0.7* gene retained in the fused ORF is not required for the kinase activity but might be important for the transcriptional shut-off (31).

**Figure 2.**
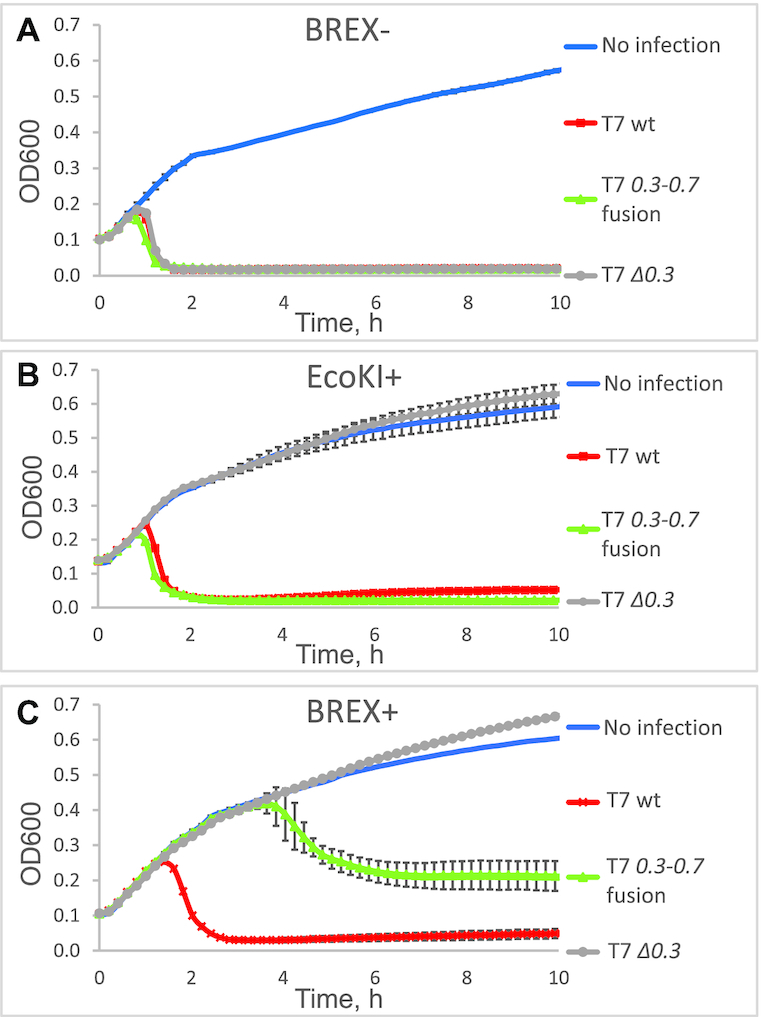
Gene *0.3* is required for productive infection of BREX+ culture by bacteriophage T7. Growth curves of BREX- (**A**), EcoKI+ (**B**) and BREX+ (**C**) cell cultures infected with wild-type, *0.3–0.7* fusion, or *Δ0.3* T7 phages at MOI = 0.001 are shown. (‘BREX-’ is an *Escherichia coli* BW25113 with an empty pBTB-2 vector; ‘BREX+’ carries a full *E. coli* HS BREX gene cluster inserted into the pBTB-2 vector; and ‘EcoKI+’ is *E. coli* AB1157 carrying a genomic copy of type I EcoKI R–M system). Phage was added at *t* = 0, and each growth curve represents the mean optical density and standard deviation values obtained from three independent experiments.

**Figure 3.**
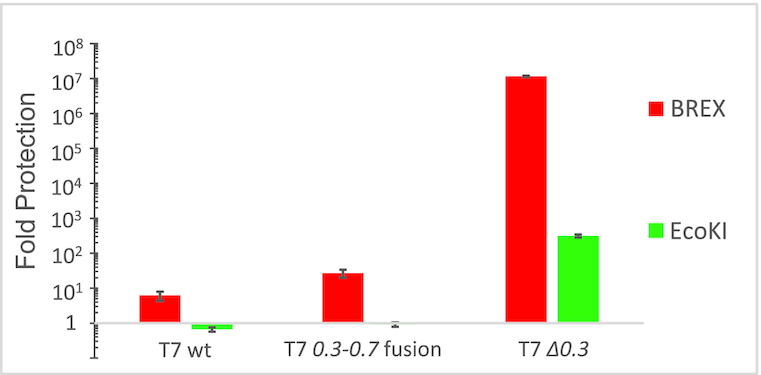
Levels of protection against T7 and its derivatives afforded by BREX and EcoKI systems. Level of protection is shown as the ratio between the titers of lysates of indicated phages obtained on non-restrictive (BREX–) relative to restrictive (BREX+ or EcoKI+) strains. In the case of the T7 *Δ0.3* phage, no plaques were detected on the BREX+ lawn, and the level of protection was calculated on the basis of appearance of zones of inhibited cell growth at certain phage concentrations. Mean values and standard deviations obtained from three independent experiments are shown. Plates are presented in Supplementary Figure S3.

We hypothesized that: (i) intact *0.3* gene is required for overcoming BREX defence during infection with wild-type T7 and (ii) the alteration to *0.3* introduced by the deletion allows the BREX system to counter the infection. To test these hypotheses, we created a T7 phage lacking the *0.3* gene (see Materials and Methods). Results of infection with the wild-type T7, the *0.3–0.7* fusion, and the *Δ0.3* phage of BREX+ and BREX- *E. coli* BW25113 cultures (carrying, respectively, a plasmid-borne BREX system or an empty vector) at a low MOI of 0.001 are presented in Figure 2. To control for Ocr function, we also infected the *E. coli* AB1157 strain, which, unlike the other strains used in the study, carries an intact type I R–M system EcoKI. All phages infected the BREX– culture with equal efficiency (lysis starting ∼1 h post-infection). Only wild-type T7 and the *0.3–0.7* fusion phage lysed the AB1157 culture, indicating that in the absence of *0.3*, EcoKI protects the cells from infection, while intact *0.3* or the *0.3–0.7* fusion are sufficient to overcome the restriction, in agreement with earlier reports (33). Wild-type T7 phage completely lysed the BREX+ culture (lysis starting ∼1.7 h post-infection), while *Δ0.3*-infected BREX+ culture showed no signs of lysis at our conditions, i.e. cells were protected by BREX from the virus. The *0.3–0.7* fusion phage showed an intermediate efficiency of infection—the infected BREX+ culture started to lyse 4 h post-infection, and the lysis appeared incomplete. The BREX+ culture was protected against T7 *Δ0.3* infection even at a higher MOI (1 or 5), i.e. in conditions where practically every cell is attacked at the beginning of the experiment by at least one phage particle (Supplementary Figure S2). The efficiency of plating (EOP) analysis,—a different assay to follow the infection process by determining the phage titer on cell lawns—revealed that, relative to the BREX– control, the BREX+ cells were marginally (∼10-fold) protected from wild-type T7, ∼100-fold protected from the *0.3*-*0.7* fusion infection, and fully protected from T7 *Δ0.3* infection (Figure 3; Supplementary Figure S3). On the AB1157 cell lawns, the EcoKI R–M system protected the cells only fromT7 *Δ0.3* infection (∼10^3^ decrease in phage titer compared to control BW25113 lawn). Overall, our findings demonstrate that wild-type Ocr allows T7 to overcome both the EcoKI and the BREX defence.

The partial anti-BREX activity of the *0.3–0.7* fusion phage could be due to the presence of the gp0.7 moiety in the fused protein, which might interfere with the function of the Ocr moiety. During prolonged propagation of the *0.3–0.7* fusion phage on BREX+ cells, we isolated multiple phage mutants that could efficiently lyse BREX+ cells (Supplementary Figure S4). All of these mutants had shortened versions of the *0.3* gene, which had arisen either due to the appearance of stop codons at the beginning of the *0.7* part of the fused *0.3–0.7* ORF or due to the duplication of a locus flanked by 11-bp direct repeats AAGTCGCACGA. Phage genomes that result from this duplication maintain the fused *0.3–0.7* ORF and acquire an additional ectopic version of *0.3* encoding 110 amino acids of Ocr fused to 11 additional amino acids (Supplementary Figure S1B). The ability of latter mutants to lyse BREX culture demonstrates that similar to the situation with the type I R–M systems (34), the last seven amino acids of Ocr are dispensable for anti-BREX defence.

### Ocr expression from plasmid shuts off the BREX defence

**Figure 4.**
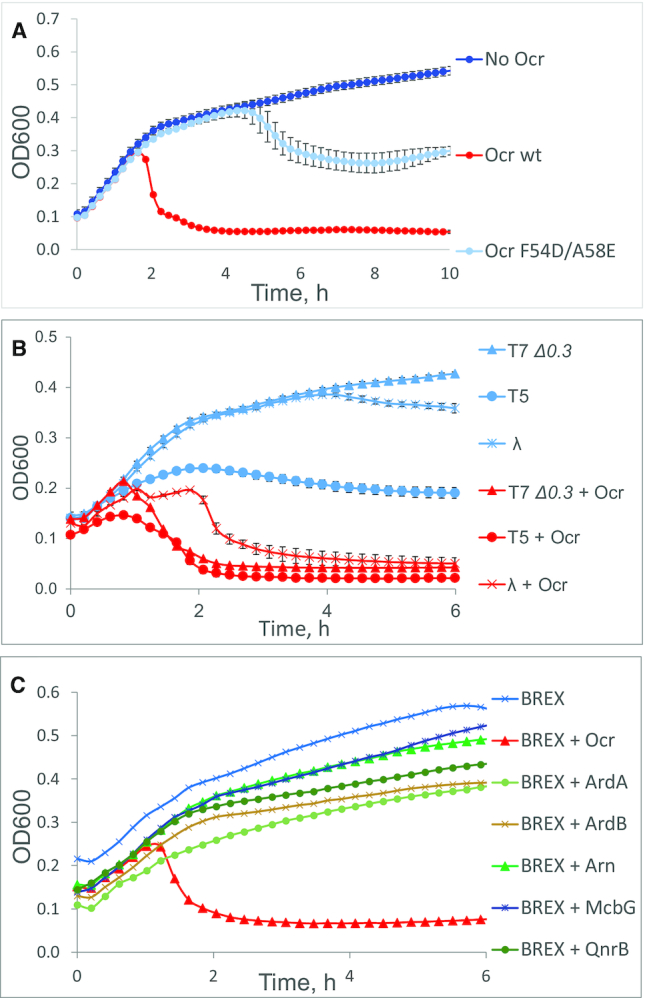
Ocr—but not other DNA mimic or antirestriction proteins—is sufficient for shutting off BREX defence by different phages. (**A**) Growth curves of BREX+ cells overproducing the indicated variants of Ocr infected with T7 *Δ0.3* at MOI = 0.001. (**B**) Growth curves of BREX+ cells overproducing wild-type Ocr infected with the indicated phages at MOI = 0.001 (T7 *Δ0.3* and T5) or MOI = 1 (λ cI857). (**C**) Growth curves of BREX+ cells expressing indicated proteins and infected with T7 *Δ0.3* at MOI = 0.001. ArdA: a DNA mimic protein from ColIb-P9 plasmid and an inhibitor of R–M I systems; ArdB: a non DNA mimic inhibitor of RM I systems from pKM101; Arn: a DNA mimic from phage T4, and an inhibitor of RM IV systems; McbG, QnrB: DNA mimic proteins of the pentapeptide repeat proteins family, inhibitors of DNA gyrase. For inducing plasmid-borne genes, cultures were grown in the presence of 13.3 mM l-arabinose (Ocr, McbG and QnrB) or 1 mM IPTG (ArdA, ArdB and Arn). Phage was added at *t* = 0. Each growth curve represents the mean optical density and standard deviations values obtained from three independent experiments. Standard deviations are not shown in panel C for the sake of clarity.

To determine whether Ocr is sufficient for overcoming BREX protection, we cloned the *0.3* gene into a pBAD L24 inducible expression vector. The plasmid was introduced into BW25113 BREX-, BW25113 BREX+ and AB1157 (BREX–, EcoKI+) cells, and the transformed cultures were infected with T7 *Δ0.3* at MOI = 0.001. In the absence of the arabinose inducer, the BREX+ and AB1157 cultures were protected from infection, as expected (data not shown). In the presence of the inducer, suppression of BREX defence was observed, with infected BREX+ culture lysing ∼2 h post-infection (Figure 4A). The extent of suppression (determined as the time of start of lysis of infected cultures) increased with increasing concentration of the inducer, suggesting that inhibition of BREX defence by Ocr is stoichiometric (Supplementary Figure S5). Expression of wild-type plasmid-borne *0.3* also protected the T7 *Δ0.3* phage from EcoKI restriction by the AB1157 culture (Supplementary Figure S6). Since a dimer of Ocr is required to mimic a bend in the DNA, we tested Ocr F54D/A58E, a previously described mutant with diminished efficiency of dimerization (35). Expression of this Ocr variant caused infected BREX+ culture to begin lysing about 5 h post-infection, and only partial lysis was observed.

The inhibition of BREX protection by Ocr was not limited to T7 infection, since BREX-sensitive phages T5 and λ acquired the ability to lyse BREX+ cultures in the presence of Ocr (Figure 4B).

We next tested the ability of several known antirestriction and DNA mimic proteins unrelated to Ocr to affect BREX protection. The proteins tested included ArdA, a DNA-mimic type I R–M system inhibitor encoded by the ColIb-P9 plasmid (36); ArdB, a non-DNA-mimic type I R–M system inhibitor encoded by the pKM101 plasmid (37); Arn, a DNA-mimic phage T4 protein that inhibits methylation-specific type IV R–M systems (38); and pentapeptide repeat family proteins McbG and QnrB, which protect cells from DNA gyrase poisons and have been suggested to possess DNA-mimicking features (39). While overproduction of some of these proteins affected cell growth, none of them affected the ability of BREX to exclude T7 *Δ0.3* infection (Figure 4C). Expression of ArdA and ArdB inhibited EcoKI restriction, as expected (Supplementary Figure S7). Thus, DNA mimicry, on its own, is not sufficient for inhibition of the BREX system by Ocr.

### Ocr directly interacts with the methyltransferase BrxX

**Figure 5.**
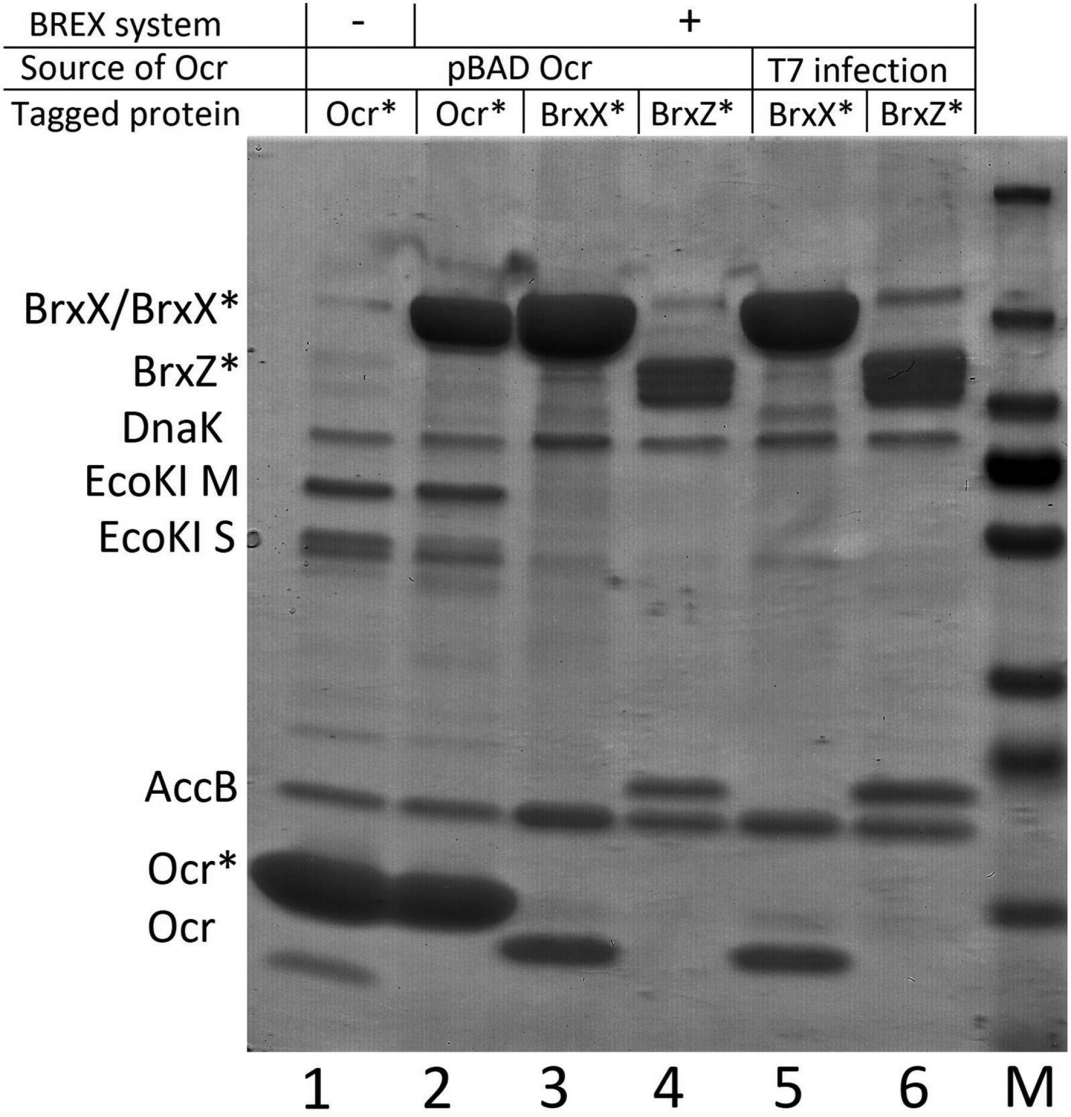
Ocr interacts with the methyltransferase BrxX. A 4–20% gradient SDS gel showing the results of pull-downs from extracts of *E. coli* EcoKI R–M+ strain BW25113 expressing, from compatible plasmids, Ocr (with or without a Strep-tag) and BREX (with or without Strep- tags on BrxX or BrxZ). Lane 1: Ocr-Strep pulled down from BREX- cells; lane 2: Ocr-Strep pulled down from BREX+ cells; lane 3: BrxX-Strep pulled down from BREX+ cells producing Ocr; lane 4: BrxZ-Strep pulled down from BREX+ cells producing Ocr; lane 5: BrxX-Strep pulled down from cells infected with wild-type T7 at MOI = 1; lane 6: BrxZ-Strep pulled down from cells infected with wild-type T7 at MOI = 1; M: PageRuler Prestained Plus ladder. Tagged proteins are labelled with an asterisk. Proteins DnaK and AccB were detected in all tested samples and thus must be non-specifically binding to the affinity resin and/or overexpressed proteins.

The results presented in the previous section suggest that Ocr might specifically interact with one of the BREX proteins. To test this idea, we performed pull-down experiments by using extracts of BW25113 (EcoKI R–M+) cells carrying the pBREX AL plasmid and a compatible plasmid overproducing C-terminally Strep-tagged Ocr. The tagged Ocr was functional as judged by its ability to overcome both BREX and EcoKI protection during T7 *Δ0.3* infection (data not shown). Previous *in vitro* studies have reported the binding of Ocr to the EcoKI methylation complex M_2_S_1_ (18,20,40). We, therefore, expected to detect the association of the EcoKI M and S subunits with Strep-tagged Ocr in BW25113 extracts. Proteins associated with Ocr were resolved by SDS-PAGE (Figure 5) and identified by MALDI-TOF mass spectrometry. EcoKI M and S subunits were detected in both BREX− (lane 1) and BREX+ (lane 2) BW25113 samples after affinity chromatography. In the case of BREX+ cells, proteins associated with immobilized Ocr included, in addition to EcoKI polypeptides, the methyltransferase BrxX (lane 2). No other BREX protein was detected.

To validate the BrxX–Ocr interaction, plasmid-borne versions of a BREX system encoding C-terminally Strep-tagged BrxB, BrxX, BrxZ or BrxL were created. The tagged versions protected the cells from phage infection (data not shown) and were, therefore, functional. A compatible plasmid expressing untagged Ocr was introduced into cells harboring each of the tagged BREX system plasmids. Then, a pull-down experiment was performed after induction of *0.3* expression. The results are presented in Figure 5 for BrxX (lane 3) and BrxZ (lane 4) and in Supplementary Figure S8 for BrxB and BrxL. As can be seen, Ocr was co-purified with BrxX but not with the other BREX system components tested.

To study the association of Ocr with BREX in a biologically relevant context, we infected cells harboring the BREX system with tagged BrxX or BrxZ with wild-type T7 phage at a high MOI. The cells were harvested 10 min after infection, disrupted, and subjected to affinity chromatography. The results showed that Ocr synthesized in the course of infection associated with BrxX (Figure 5, lane 5) but not with BrxZ (lane 6), in agreement with the results obtained in the absence of phage infection. Overall, our findings demonstrate that Ocr binds BrxX. We assume that this binding is responsible for the ability of Ocr to overcome BREX defence.

### Ocr decreases BREX methylation

**Figure 6.**
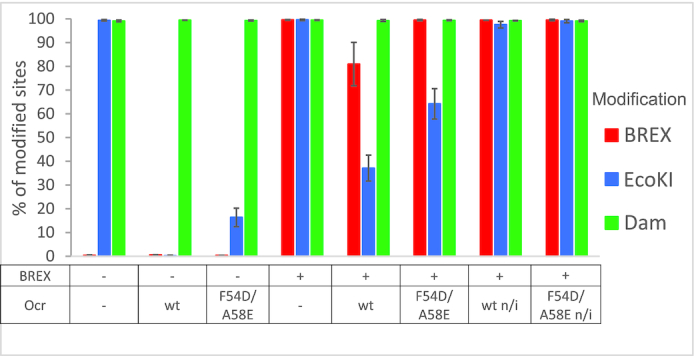
Effect of Ocr on adenine-specific methylation of *E. coli* genome. Interpretation of the Pacific Bioscience sequencing data in a form of percentages of modified sites (BREX, EcoKI and Dam) in the genomes of BW25113 (EcoKI R– M+), BREX- and BREX+ strains expressing, where indicated, wild-type or mutant F54D/A58E Ocr. Shown are mean values and standard deviations obtained by using DNA prepared from three independent cultures. A site was treated as modified if it's modification QV exceeded 30. Data, demonstrating modification QV and IPD ratios for all samples, are presented in Supplementary Figure S9.

Since Ocr was shown to bind the BrxX methyltransferase, we wondered if it affects BREX methylation *in vivo*. The methylation state of BREX sites (GGTAAG) in the genome of *E. coli* BW25113 cells harboring the pBREX AL plasmid or control empty vector and a compatible Ocr expression plasmid was determined (Supplementary Table S4). After 1 h of growth in LB medium, Ocr production was induced in both the control and BREX+ cultures for 2 h. Within this period, BREX system became inactivated, as evidenced by efficient infection of the cells with the T7 *Δ 0.3* phage. Genomic DNA was prepared from induced and non-induced cultures and subjected to PacBio sequencing, which detects the methylation state of DNA. The proportions of Dam (GATC) and EcoKI (GCACNNNNNNGTT and AACNNNNNNGTGC) specific methylation sites in cells with and without Ocr were also determined (Supplementary Tables S5 and S6). Sites with QV value >30 were considered as modified. In cells lacking the Ocr expression plasmid, ∼100% of Dam and EcoKI sites were methylated (Figure 6). In the presence of the BREX plasmid, BREX sites also became methylated. Ocr did not affect methylation of the Dam sites but completely abolished the methylation of EcoKI sites in BREX- cells, as expected. Strikingly, most (80%) BREX sites remained methylated in the presence of Ocr in BREX+ cells. The inhibition of EcoKI methylation by Ocr in BREX+ cells was less pronounced (∼40% of EcoKI sites remained methylated) than in BREX- cells, which might be explained by competitive binding of limiting quantities of Ocr to both EcoKI and BREX systems components. Despite partial methylation of BREX sites, BREX+ cells expressing Ocr grew normally, suggesting that the presence of unmethylated sites did not trigger a suicidal response. The positions of non-methylated BREX sites varied among biological replicas (Supplementary Table S7, Supplementary Figure S10), suggesting that they primarily appeared due to a stochastic rather than deterministic process.

PacBio sequencing of DNA from BREX- cells expressing Ocr F54D/A58E showed that the mutant was less efficient in inhibition of EcoKI methylation than wild-type Ocr (∼16% residual methylation versus ∼0% for wild-type Ocr). In BREX+ cells expressing the mutant Ocr, 60% of EcoKI sites were methylated (compared with ∼40% methylation in cells expressing wild-type Ocr), while nearly all BREX sites remained methylated.

**Figure 7.**
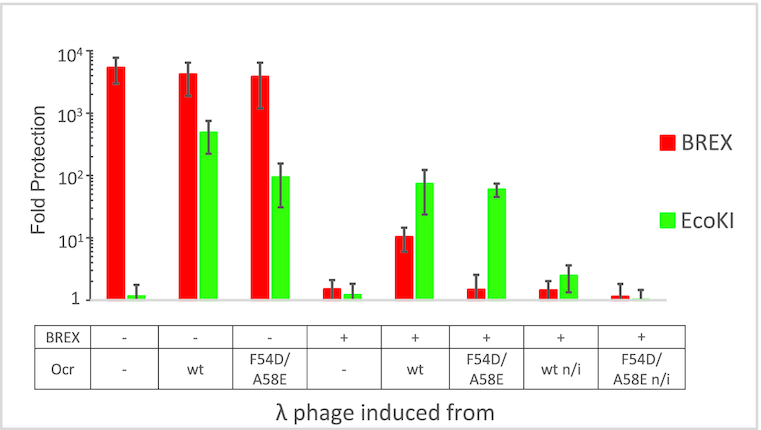
Phage λ induced from BREX+ culture producing Ocr overcomes BREX defence. λ cI857 was induced from lysogenic BREX– or BREX+ strains that overproduced, where indicated, wild-type Ocr or mutant F54D/A58E Ocr. Phage lysates were titrated on BREX–, BREX+ and EcoKI+ cell lawns. The level of protection is shown as the ratio of phage titers obtained on non-restrictive (BREX-) compared to restrictive (BREX+ or EcoKI+) hosts. Decreased BREX protection against phage λ induced from BREX+ or BREX + Ocr strains indicate the presence of BREX-specific modifications. Plates showing phage titer measurements are presented in Supplementary Figure S11.

The effect of Ocr on methylation of BREX sites was also assessed by a λ prophage induction assay (7) (Figure 7; Supplementary Figure S11). Introduction of a BREX plasmid into a λ lysogenic strain leads to the production of modified induced phage progeny that can overcome BREX defence and infect BREX+ cells as efficiently as the BREX- cells. Phages induced from BREX– cells infect BREX+ cells inefficiently (∼10^4^-fold protection). The presence of wild-type Ocr in BREX- lysogens did not affect the ability of induced phages to infect the BREX+ cells, however, it made them sensitive to EcoKI restriction by the AB1157 cells (∼10^3^-fold protection), indicating that the EcoKI methylation was inhibited in the strain used for prophage induction. Phages induced from BREX+ lysogens in the presence of wild-type Ocr cannot productively infect AB1157 cells (∼10^2^-fold protection) and show only partial resistance to BREX (∼10^1^-fold protection). Phages induced from the BREX+ culture producing Ocr F54D/A58E infected the BREX+ cells productively, suggesting complete modification of their DNA, consistent with the PacBio data. The level of BREX protection against phages induced from BREX+ lysogens was positively correlated with the concentration of l-arabinose used to activate the plasmid-borne *0.3* gene expression (Supplementary Figures S12 and S13). The results thus indicate that increasing concentrations of Ocr lead to a gradual decrease in the levels of phage DNA modification, which, in turn, increases the level of BREX defence.

## DISCUSSION

The continuous arms race between bacteria and phages ensures that, for every cellular defensive system of a cell, there is a viral mechanism that can overcome it. Known examples include various antirestriction proteins and, more recently, a plethora of anti-CRISPR proteins targeting CRISPR effectors of various types (41). As new cellular defence mechanisms are discovered (42–44), it is only a matter of time before viral systems that overcome them are revealed. In this work, we have established that T7 Ocr, a DNA mimic protein that inhibits type I R–M systems, also abrogates the protective function of the BREX system. Ocr interacts with the M.EcoKI and R.EcoKI complexes, inhibiting both restriction and modification activities of the system. In the case of BREX, Ocr specifically binds to BrxX, a DNA methyltransferase, which is responsible for the epigenetic modification of BREX sites required for self versus non-self differentiation. In the presence of wild-type Ocr produced from a plasmid, EcoKI methylation was lost at almost 65% of the sites, while only ∼20% of BREX sites lost methylation. In the case of EcoKI, the viability of cells with the partially methylated genome was not affected since Ocr also prevents the ability of restriction complexes to bind and/or cleave DNA. Because cells with partially methylated BREX sites grow as well as BREX- cells or cells with fully modified DNA, it is highly likely that Ocr inhibits not just the BrxX methyltransferase but also the yet to be defined complex that is responsible for the exclusion of the phage. It therefore follows that this complex contains BrxX. This is similar to the type I R–M restriction complexes, which are composed of the methylation complex and an additional restriction subunit.

Previous studies have described Ocr mutants that tightly bind M.EcoKI and inhibit restriction without affecting methylation *in vivo* and *in vitro* (34). We investigated one such mutant—a dimerization-defective F54D/A58E (35)—and found that it inhibited BREX defence without affecting the methylation state of the host genome. A similar effect was observed with wild-type Ocr at conditions of reduced expression. These data suggest that Ocr might be also preferentially inhibiting BREX exclusion complexes.

Being a DNA mimic, Ocr can potentially interact not just with type I R–M or BREX components, but also with other DNA-binding proteins. Indeed, it was shown to relieve H-NS-related silencing (38) and inhibit host RNA Polymerase in specific conditions (23). The effect of Ocr on BREX appears to be specific, because other DNA mimicking proteins do not overcome BREX defence. The particular features of Ocr that underlie this specificity would require structural analysis of its interactions with BREX, which is currently ongoing in our laboratory.

## Supplementary Material

gkaa290_Supplemental_Files
