# Phage T7 DNA mimic protein Ocr is a potent inhibitor of BREX defence

**DOI:** 10.1093/nar/gkaa510

**Published:** 2020-06-09

**Authors:** Artem Isaev, Alena Drobiazko, Nicolas Sierro, Julia Gordeeva, Ido Yosef, Udi Qimron, Nikolai V Ivanov, Konstantin Severinov

**Affiliations:** Skolkovo Institute of Science and Technology, Moscow 143028, Russia; Skolkovo Institute of Science and Technology, Moscow 143028, Russia; Philip Morris International R&D, Philip Morris Products S.A., Neuchatel 2000, Switzerland; Skolkovo Institute of Science and Technology, Moscow 143028, Russia; Department of Clinical Microbiology and Immunology, Sackler Faculty of Medicine, Tel Aviv University, Tel Aviv 69978, Israel; Department of Clinical Microbiology and Immunology, Sackler Faculty of Medicine, Tel Aviv University, Tel Aviv 69978, Israel; Philip Morris International R&D, Philip Morris Products S.A., Neuchatel 2000, Switzerland; Skolkovo Institute of Science and Technology, Moscow 143028, Russia; Waksman Institute of Microbiology, Piscataway, NJ 08854, USA; Institute of Gene Biology, Russian Academy of Sciences, Center for Precision Genome Editing and Genetic Technologies for Biomedicine, Institute of Gene Biology, Russian Academy of Sciences, 34/5 Vavilov str., 119334 Moscow, Russia

*Nucleic Acids Research*, 2020, 48(10): 5397–5406, https://doi.org/10.1093/nar/gkaa290

In Figure 4C, the authors inadvertently omitted the growth curve for BREX + ArdB.

**Figure 4.**
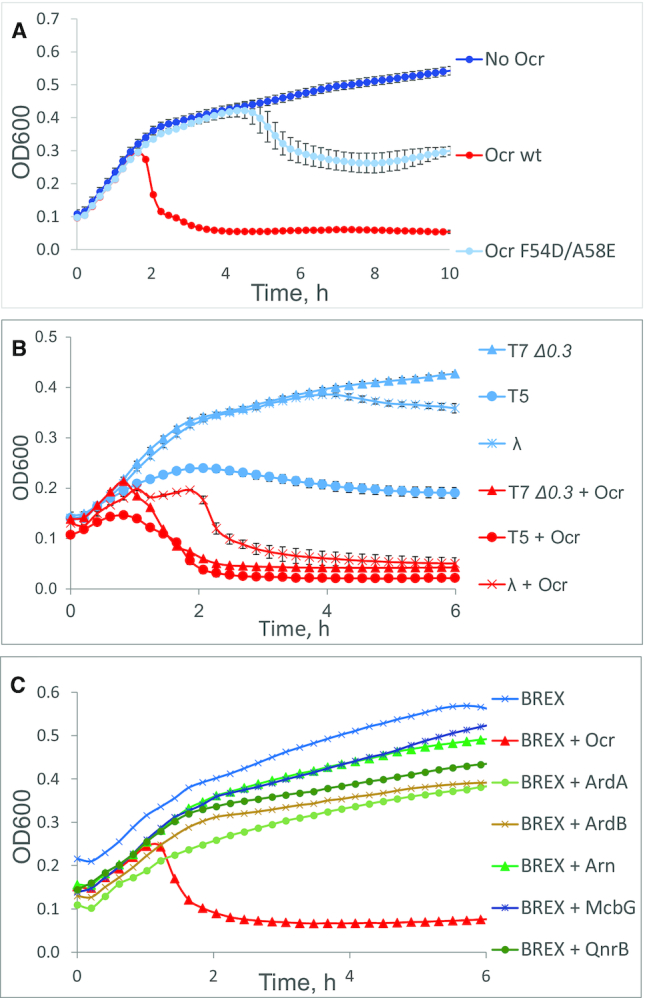
Ocr––but not other DNA mimic or antirestriction proteins––is sufficient for shutting off BREX defence by different phages. (**A**) Growth curves of BREX+ cells overproducing the indicated variants of Ocr infected with T7 Δ0.3 at MOI = 0.001. (**B**) Growth curves of BREX+ cells overproducing wild-type Ocr infected with the indicated phages at MOI = 0.001 (T7 Δ0.3 and T5) or MOI = 1 (λ cI857). (**C**) Growth curves of BREX+ cells expressing indicated proteins and infected with T7 Δ0.3 at MOI = 0.001. ArdA: a DNA mimic protein from ColIb-P9 plasmid and an inhibitor of R–M I systems; ArdB: a non DNA mimic inhibitor of RM I systems from pKM101; Arn: a DNA mimic from phage T4, and an inhibitor of RMIV systems; McbG, QnrB: DNA mimic proteins of the pentapeptide repeat proteins family, inhibitors of DNA gyrase. For inducing plasmid-borne genes, cultures were grown in the presence of 13.3 mM Larabinose (Ocr, McbG and QnrB) or 1mMIPTG (ArdA, ArdB and Arn). Phage was added at t = 0. Each growth curve represents the mean optical density and standard deviations values obtained from three independent experiments. Standard deviations are not shown in panel C for the sake of clarity.

A new figure is provided below, and the published article has been updated.

This error does not affect the results or conclusion of the article.

