# Supplementary material for: Phage T7 DNA mimic protein Ocr is a potent inhibitor of BREX defence
Source: Nucleic Acids Res. 2020 Apr 27;48(10):5397–406. doi: 10.1093/nar/gkaa290 (PMC7261183; doi:10.1093/nar/gkaa290)

**Supplementary Table S1. Bacterial strains, plasmids, and phages used in the study.**

| ***E. coli* strain** | **Comments** | **Source** |
| --- | --- | --- |
| BW25113 | *E. coli* K12 F^–^ *Δ*(*araD-araB*)567 *ΔlacZ*4787(::*rrnB*-3) λ– *rph*-1 *Δ*(*rhaD-rhaB*)568 *hsdR*514 | Lab stock |
| AB1157 | *E. coli* K12 F^–^ *thr-*1 *araC*14 *leuB*6(Am) *Δ(gpt-proA)*62 *lacY*1 *tsx-*33 *qsr'-*0 *glnX*44(AS) *galK*2(Oc) λ^-^ *Rac-*0 *hisG*4(Oc) *rfbC*1 *mgl-*51 *rpoS*396(Am) *rpsL*31 *kdgK*51 *xylA*, *mtl-*1 *argE*3(Oc) *thiE*1, Str^R^ | Lab stock |
| XL1-Blue | *E. coli* K12 *recA*1 *endA*1 *gyrA*96 *thi*-1 *hsdR*17 *supE*44 *relA*1 *lac* [*F'proAB lacI^q^ZΔM*15 *Tn*10], Tet^R^ | Evrogen |
| NEB5α | *E. coli* K12 *huA*2 (*argF-lacZ*)*U*169 *phoA* *glnV*44 80 (*lacZ*)*M*15 *gyrA*96 *recA*1 *relA*1 *endA*1 *thi-*1 *hsdR*17 | NEB |
| BW25113 λ_ts_ lysogen | λ *cI*_857_ *bor::Cm,* Cm^R^ | (7) |
| BW25113 Δ*trxA* | *trxA::Kn*, Kn^R^ | KEIO |
| T7 express (BL21) | *E. coli* B F^–^ λ– *fhuA*2 *lacZ::*T7 *gene1* [*lon*] *ompT gal sulA*11 *R*(*mcr*73*::miniTn*10*--Tet^S^* )2 [*dcm*] *R*(*zgb-*210*::Tn*10*--Tet^S^* ) *endA*1 *D*(*mcrCmrr*)114*::IS*10 | NEB |
| **Phage** | **Comments** | **Source** |
| T7 wt |  | Lab stock |
| T7 *0.3-0.7* fusion | *Δ*1256-2735 | This work |
| T7 *0.3-0.7* fusion reversion | List of T7 mutants wit *0.3-0.7* fusion reversion selected after growth on BREX+ culture might be found in Supp. figure 4A | This work |
| T7 *Δ0.3* | *0.3*::*trxA* | This work |
| T5 |  | Lab stock |
| λ_vir_ | λ mutant with an obligatory lytic lifecycle | Lab stock |
| **Plasmids** | **Comments** | **Source** |
| pUC-0.3 | Gene *trxA* flanked with regions surrounding T7 *0.3*, pUC-19, Amp^R^ | This work |
| pBAD L24 | pBAD/His B *Δ312-455* (lacks features for protein purification) with introduced EcoRI and SacI restriction sites, Amp^R^ | Lab stock |
| pBAD Ocr wt | pBAD L24 with T7 Ocr, araBAD promoter, Amp^R^ | This work |
| pBAD Ocr F54D/A58E | pBAD L24 with T7 Ocr F54D/A58E, araBAD promoter, Amp^R^ | This work |
| pBAD Ocr C-strep | pBAD L24 with T7 Ocr carrying C-terminal strep-tag, araBAD promoter, Amp^R^ | This work |
| pBTB-2 | Kn^R^ | (7) |
| pBREX AL | 6 genes BREX cluster from *E. coli* HS in low copy number vector pBTB-2, Kn^R^ | (7) |
| pBREX AL BrxB C-Strep | Strep-tagged *brxB* in a context of full BREX cluster, Kn^R^ | This work |
| pBREX AL BrxX C-Strep | Strep-tagged *brxX* in a context of full BREX cluster, Kn^R^ | This work |
| pBREX AL BrxZ C-Strep | Strep-tagged *brxZ* in a context of full BREX cluster, Kn^R^ | This work |
| pBREX AL BrxL C-Strep | Strep-tagged *brxL* in a context of full BREX cluster, Kn^R^ | This work |
| pOcr F54D/A58E | pUC-19, Ocr with diminished dimerization efficiency, Amp^R^ | (35) |
| pArdA | *ardA* from ColIb-P9 in pUC-18, lac promoter; Amp^R^ | (46) |
| pArdB | *ardB* from R64 (IncI1) in pUC-18, lac promoter; Amp^R^ | (46) |
| pArn | *arn* from T4 in pTZ57R vector, lac promoter; Amp^R^ | (45) |
| pMcbG | *mcbG* with N-6His in pBAD/His B vector, araBAD promoter; Amp^R^ | Lab stock |
| pQnrB | *qnrB* with N-6His in pBAD/His B vector, araBAD promoter; Amp^R^ | Lab stock |

**Supplementary Table S2. Primers used in the study.**

| **Name** | **Sequence (5′->3′)** |
| --- | --- |
| IY27F | GTACCATATGGCAAGGTGCCCTTTATGATATTCACTAATAACTGCACGAGGTAACACAAGGATCCGTCAGCCTGCAGTTC |
| IY27R | TAAGTCTAGATCAGACCGTATTGCACGTTGGTAGTAGACATTACTCTTCATCCTCCTCGTTGTAGGCTGGAGCTGCTTCG |
| Ocr_F | TTTGGGCTAACAGGAGGAAGAATTCATGGCTATGTCTAACATGAC |
| Ocr_R | CAGCCAAGCTTGCGGCCGCGAGCTCTTACTCTTCATCCTCCTCGT |
| Ocr_R_Strep | CAGCCAAGCTTGCGGCCGCGAGCTCTTATTTTTCGAACTGCGGGTGGCTCCACTCTTCATCCTCCTCGTACT |
| 5′ Sac I | TATGGAGCTCCTAAAGCATGTG |
| 3′ Xba I | CGTTTCTAGAACTTCACCAC |
| BrxB_R_Strep | GAACTGCGGGTGGCTCCAGCTGCCGCCGCTGCCGCCTTGAGGATTCAATGTTGC |
| BrxB_F | GGAGCCACCCGCAGTTCGAAAAAACATTGAATCCTCAATGA |
| 5′ Xba I | CGGTGGTGAAGTTCTAGAAACGAC |
| 3′ Nhe I | CAATAGCTAGCATACGAATAAGCAAC |
| BrxX_R_Strep | GAACTGCGGGTGGCTCCAGCTGCCGCCGCTGCCGCCGATCGCCTCTGGGGC |
| BrxX_F | GGAGCCACCCGCAGTTCGAAAAATAAACCAGACGGCGCGTTC |
| 5′ Nhe I | CGTTGCTTATTCGTATGCTAGCTATTG |
| 3′ Bgl II | GGTGAGATCTTTACGCACCACAC |
| BrxZ_R_Strep | GAACTGCGGGTGGCTCCAGCTGCCGCCGCTGCCGCCATTCACACCCAGCGCC |
| BrxZ_F | GGAGCCACCCGCAGTTCGAAAAATAAGCATGCAACGAATTCA |
| 5′ Not I | AAGCGCGGCCGCGAAATTC |
| 3′ Sac I | TTAGGAGCTCCATACCTG |
| BrxL_R_Strep | GAACTGCGGGTGGCTCCAGCTGCCGCCGCTGCCGCCATTCACACCCAGCGCC |
| BrxL_F | GGAGCCACCCGCAGTTCGAAAAATAAGCATGCAACGAATTCA |

**SUPPLEMENTARY FIGURES**

Supplementary figure 1. A) Organization of the left end of T7 phage genome and positions of direct repeats allowing locus 2 deletion and appearance of *0.3*–*0.7* fusion (R2, AGGAAGTCGAGG, start positions 1244 and 2724). B) Duplication of the locus 1 in the *0.3*–*0.7* fusion genome through R1 repeats (R1, AAGTCGCACGA, start positions 568 and 2736) restores the short version of the *0.3* gene.

**A**


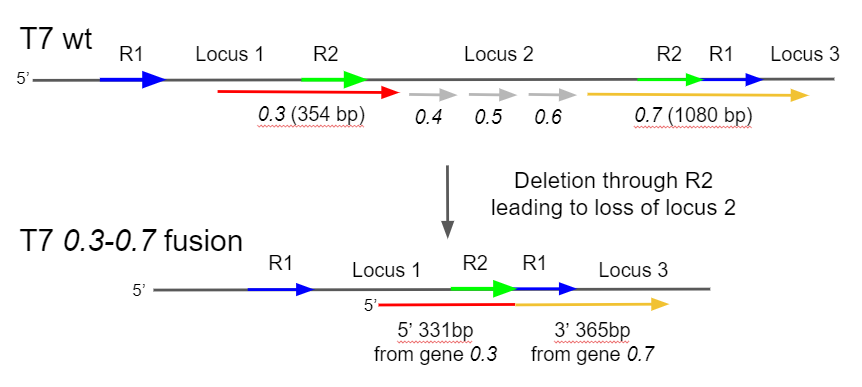


**B**


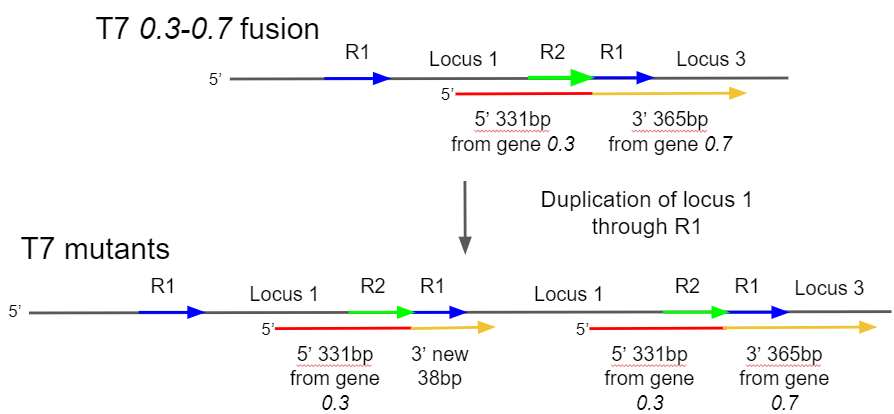


Supplementary figure 2. BREX+ culture is protected against T7 *Δ0.3* infection at high MOI. Growth curves of BREX+ and BREX- cultures infected with T7 *Δ0.3* strain at MOI = 1 or 5. Phage was added at *t=0*, and each growth curve represents the mean optical density values and standard deviations obtained from three independent experiments.

T7 *Δ0.3*, MOI=5, BREX+

T7 *Δ0.3*, MOI=1, BREX+

T7 *Δ0.3*, MOI=5, BREX-

Supplementary figure 3. Plating of T7 phage and its derivatives on BREX-, BREX+, and AB1157 (EcoKI+) cell lawns. Tenfold dilutions of T7 lysates are indicated at the bottom of each figure. Representative data from an experiment repeated three times.
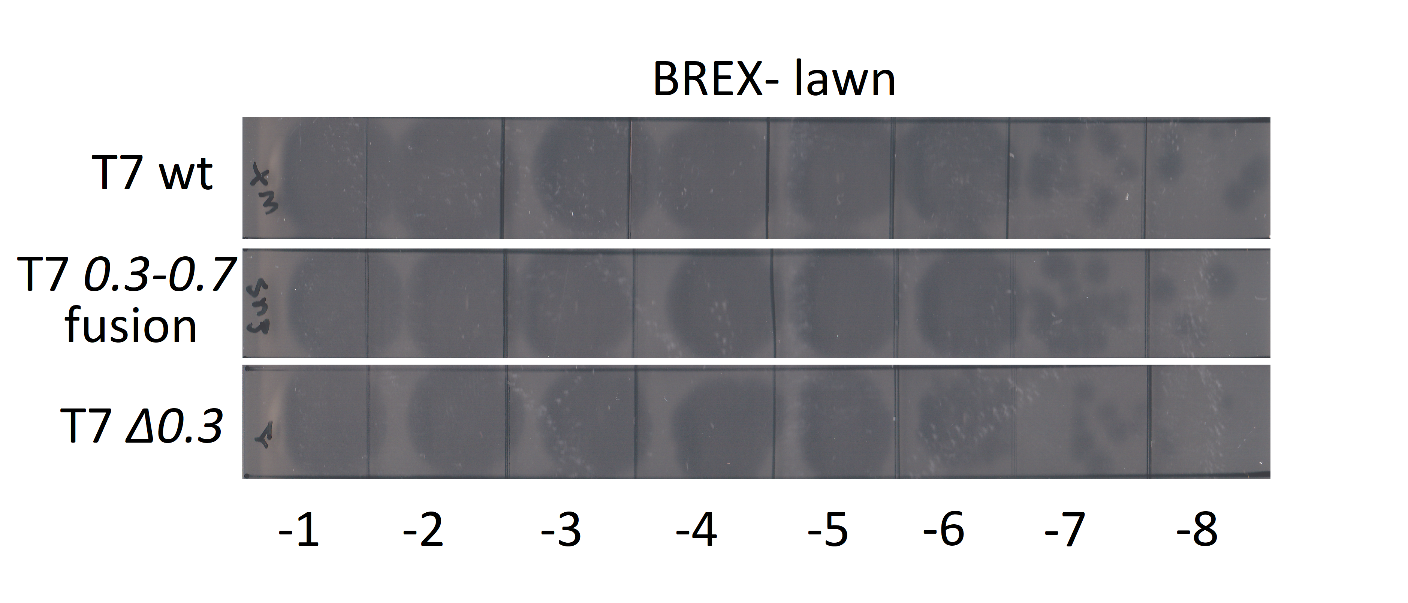


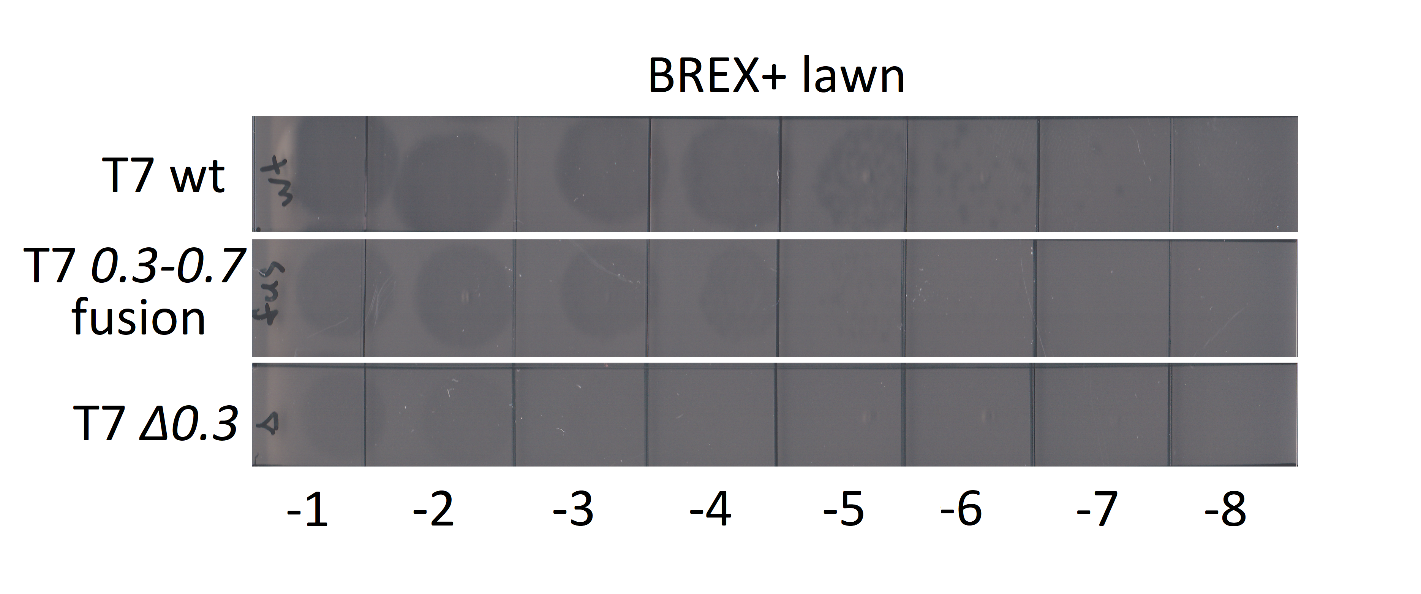


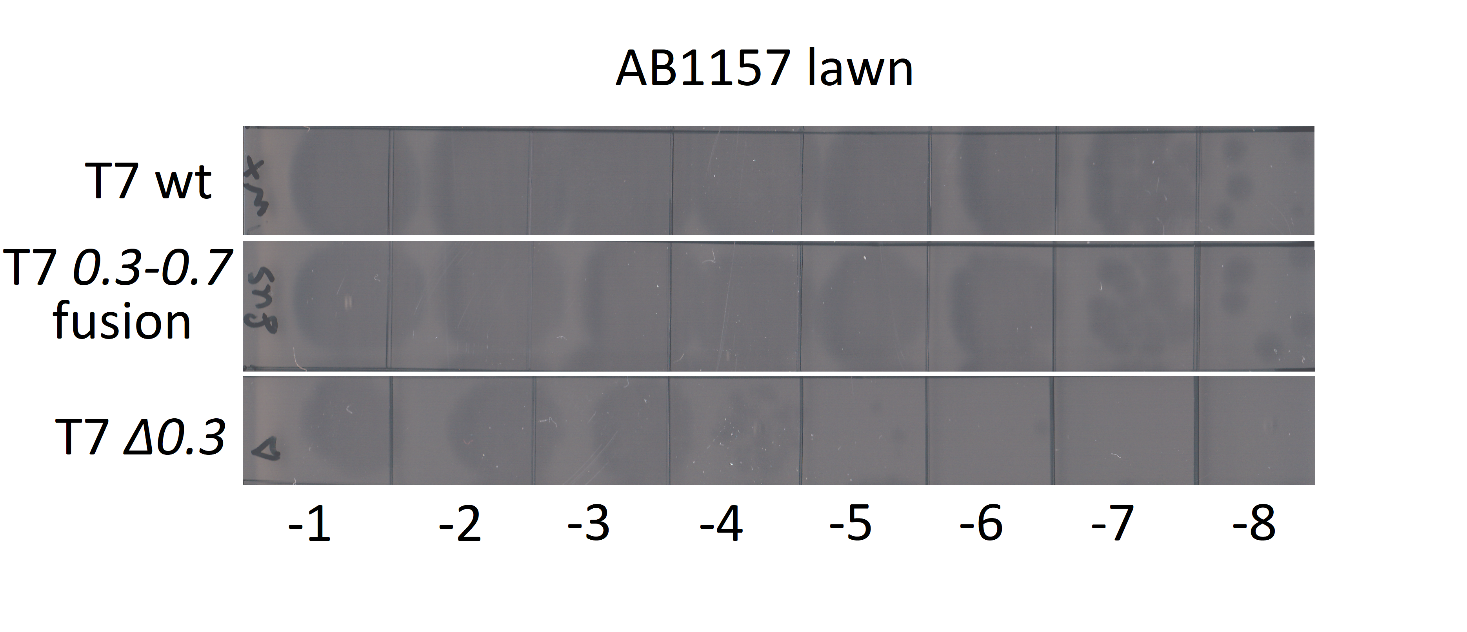


Supplementary figure 4. A) Mutations in the *0.3–0.7* fusion ORF detected after propagation of T7 *0.3–0.7* fusion phage on a BREX+ culture. Length of the resulting Ocr version in each strain is shown in column 2. Yellow color – early stop codons in the *0.3–0.7* fusion ORF. Green color – duplication of locus 1 that creates a copy of the *0.3* gene (Ocr 111 + 11 new amino acids). B) Growth curves of BREX- and BREX+ cultures infected at MOI = 0.001 with representative T7 mutants obtained in the course of T7 *0.3–0.7* fusion propagation on BREX+. Mutant 2.4 restores the short version of Ocr by an early stop codon in the *0.7* part of the fusion ORF; Mutant J3 is bearing the fusion *0.3–0.7* ORF together with the shortened version of the *0.3* gene resulting from locus 1 duplication.


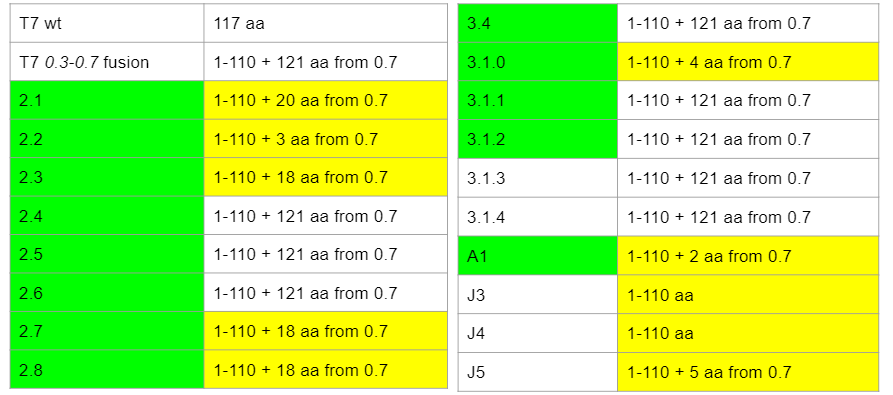


**A**

T7 *0.3-0.7* fusion, BREX-

T7 *0.3-0.7* fusion, BREX+

Supplementary figure 5. Growth curves of BREX+ cultures in the presence of pBAD Ocr wt plasmid infected with T7 *Δ0.3* phage at MOI = 0.001. Different concentrations of L-arabinose inducer are shown. Titration demonstrates an almost linear effect on the efficiency of lysis in the 67 nM to 67 μM concentration range of L-arabinose.

Supplementary figure 6. Growth curves of AB1157 (EcoKI +) cells overproducing Ocr infected with T7 *Δ0.3* at MOI = 0.001. The cultures were grown in the presence of 13.3 mM L-arabinose to induce the plasmid-borne *0.3* gene.

Supplementary figure 7. Growth curves of the AB1157 (EcoKI+) culture carrying plasmids expressing DNA mimic and anti-restriction proteins, infected with T7 *Δ0.3* phage at MOI = 0.0001. Induction of expression of plasmid-borne genes with 13.3 mM L-arabinose (Ocr) or 1 mM IPTG (ArdA, ArdB).

AB1157 + ArdB + T7 *Δ0.3*

AB1157 + ArdA + T7 *Δ0.3*

AB1157 + Ocr + T7 *Δ0.3*

Supplementary figure 8. Pull-downs of Strep-tagged BrxB – BrxX – BrxL – BrxZ with or without plasmid-borne Ocr. A 5–10% gradient SDS-PAGE. **1**,**13** – PageRuler Prestained Plus ladder; **2** – BrxB; **3** – BrxB + Ocr; **4** – BrxX; **5**,**6** – Fractions of BrxX + Ocr; **7** – BrxL; **8** – BrxL + Ocr; **9** – BrxZ; **10**,**11**,**12** – Fractions of BrxZ + Ocr. The band corresponding to Ocr was detected only in the pull-down with BrxX. Low molecular-weight bands in samples 3, 9, 10, and 12 were not identified as Ocr.


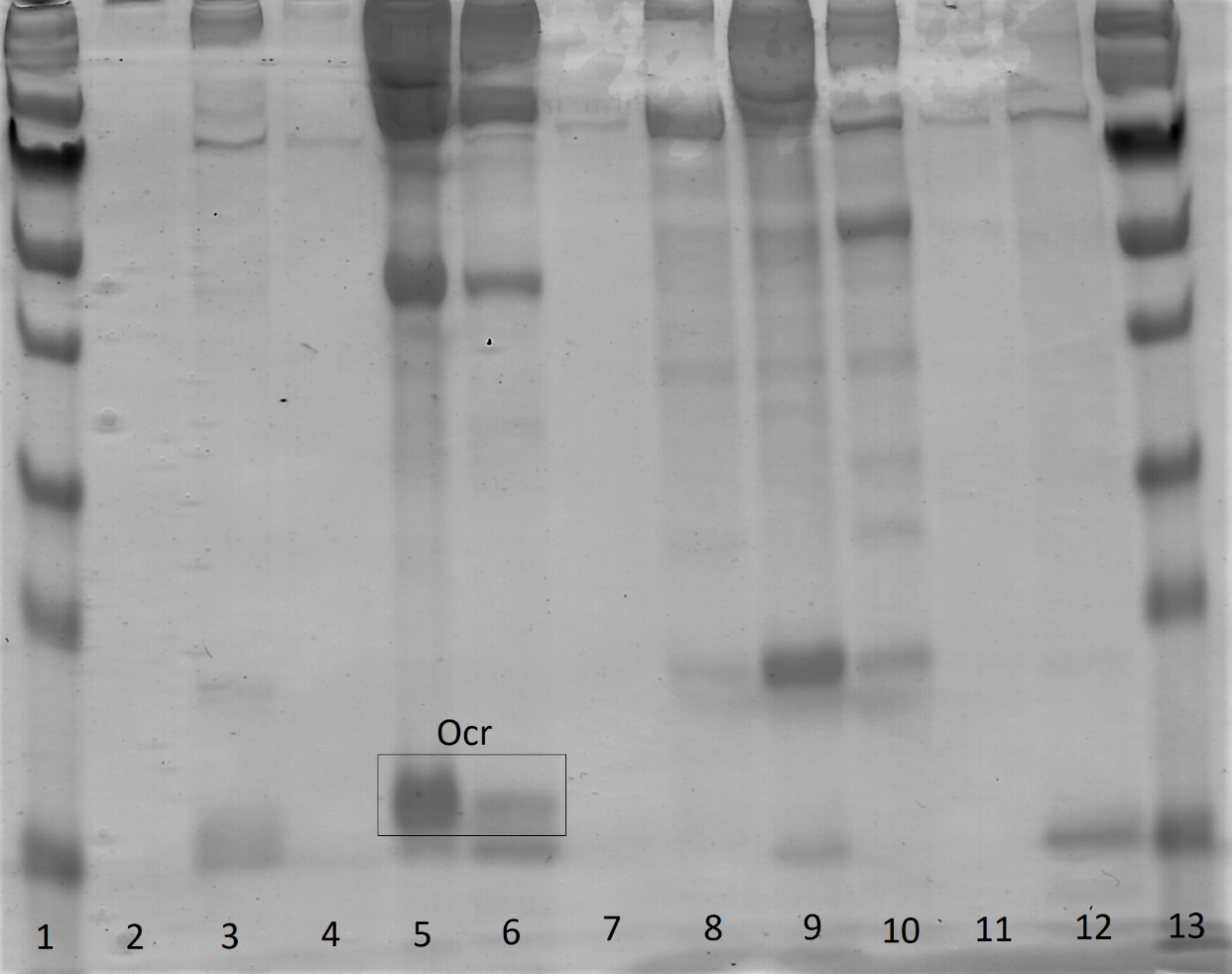


Supplementary figure 9. Effect of Ocr on adenine-specific methylation of the *E. coli* genome. Distribution of the modification QVs and of the IPD ratios for BREX (A), EcoKI (B), and Dam (C) sites in the genomes of BW25113 (EcoKI R- M+) BREX- and BREX+ cells expressing, where indicated, wild-type or mutant F54D/A58E Ocr, as revealed by Pacific Bioscience sequencing. Data obtained from experiment performed in triplicate are shown. Red lines label a modification QV of 30 and an IPD ratio of 6, which indicates a six-fold longer than expected adenine incorporation.

**A**

**B**

**C**

.


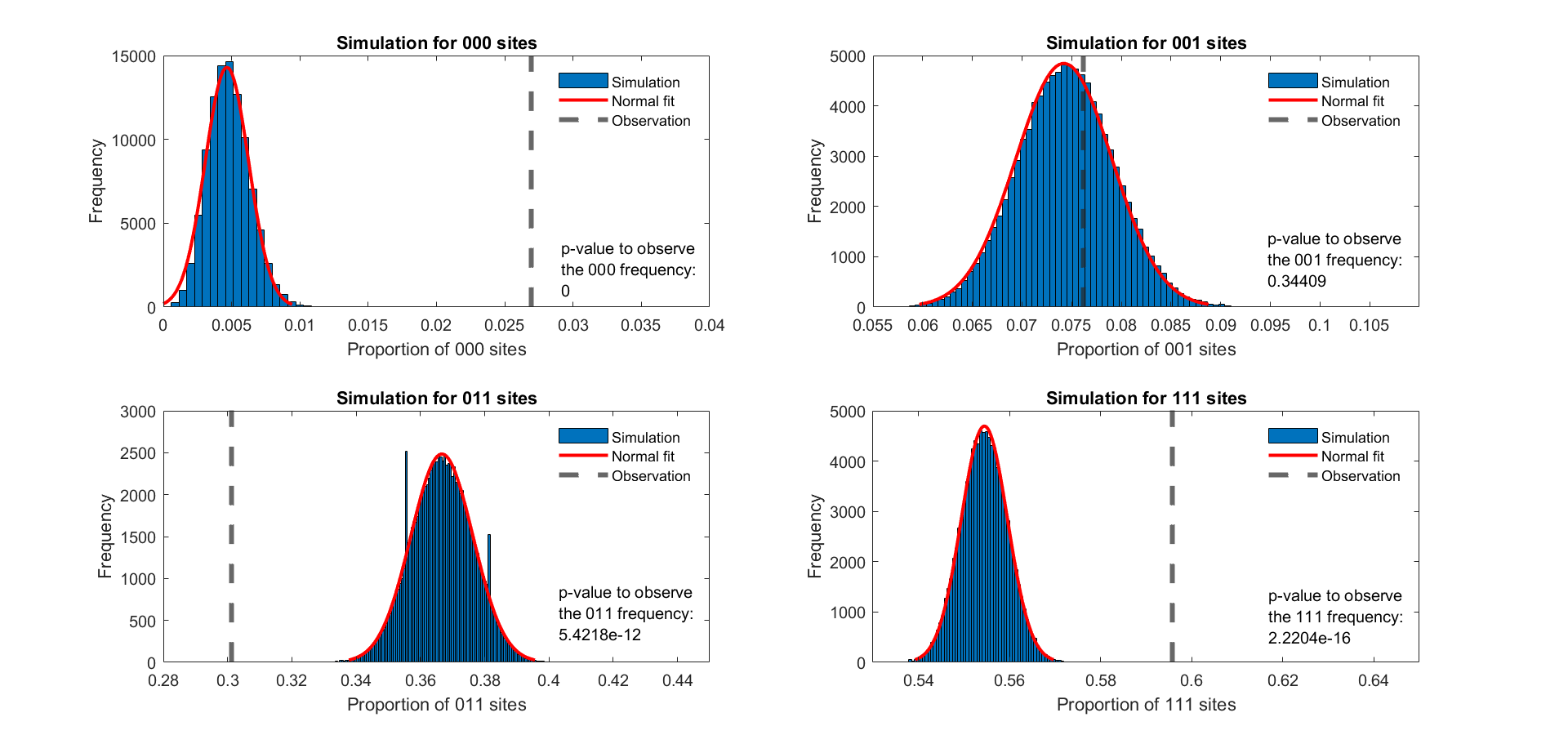
Supplementary figure 10. Co-occurrence of non-methylated BREX sites in the biological triplicates of BREX+ cultures expressing Ocr wt. 0 – non-methylated BREX site; 1 – methylated BREX site. The order of non-methylated positions for each repeat was randomly shuffled 100,000 times, and the distribution of site co-occurrences between replicas followed the normal fit. The real co-incidence between sites for each situation is marked with a dashed line. The proportion of reproducibly non-methylated positions (000 situation - 2,7%) is 5.6 times higher than what might be expected for the random distribution. Distribution of BREX sites and their modification status can be found in Supplementary table 4.

Supplementary figure 11. Plating of λ phage induced from the indicated strains (numbers on top of the plates) on BREX-, BREX+, and AB1157 (EcoKI+) cell lawns. Tenfold dilutions of λ lysates are indicated on the left. **1** – BREX-; **2** – BREX+; **3** – BREX-, Ocr wt; **4** – BREX+, Ocr wt; **5** – BREX+ Ocr wt, no induction; **6** – BREX-, Ocr F54D/A58E; **7** – BREX+, Ocr F54D/A58E, no induction; **8** - BREX+, Ocr F54D/A58E. Representative data from a triplicate experiment.


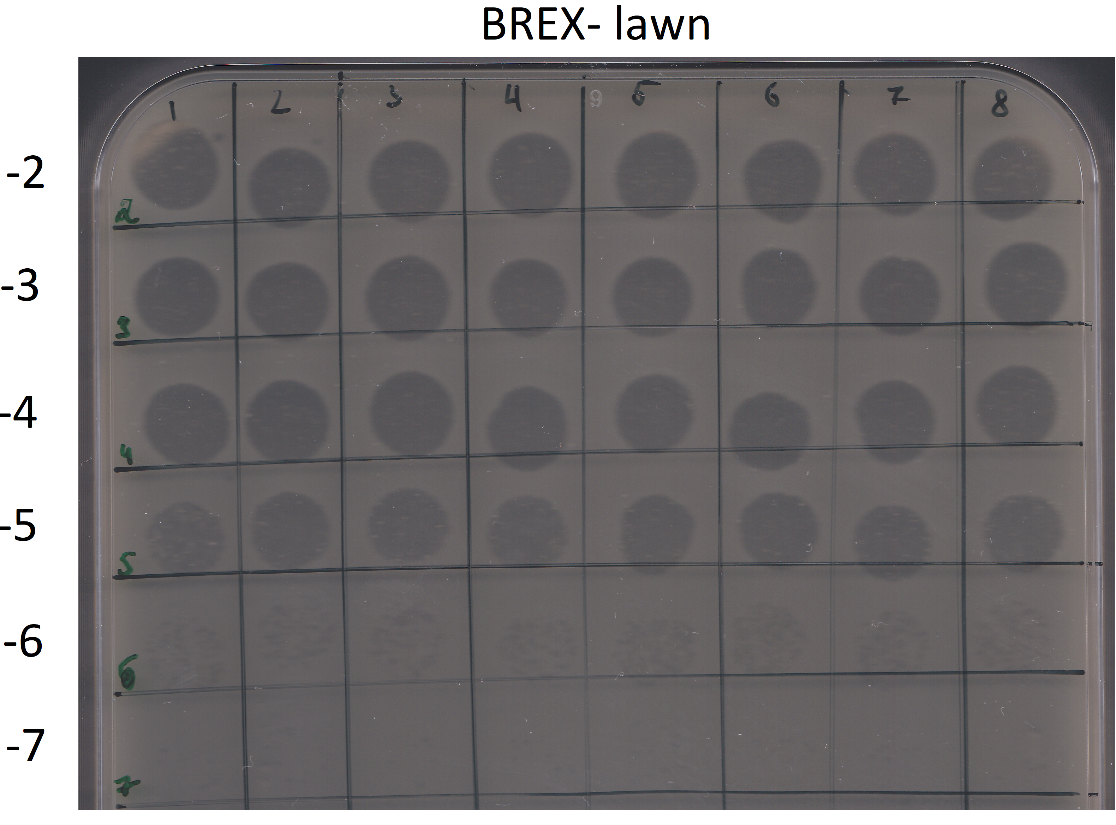


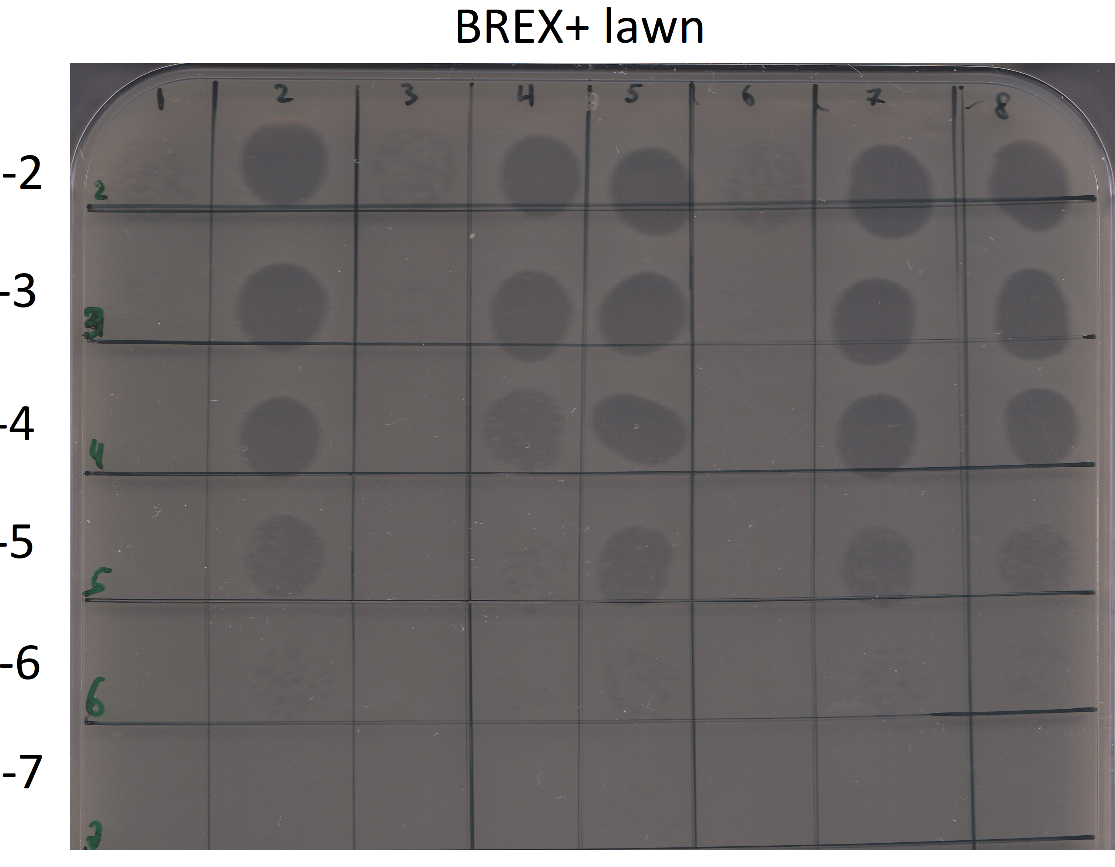


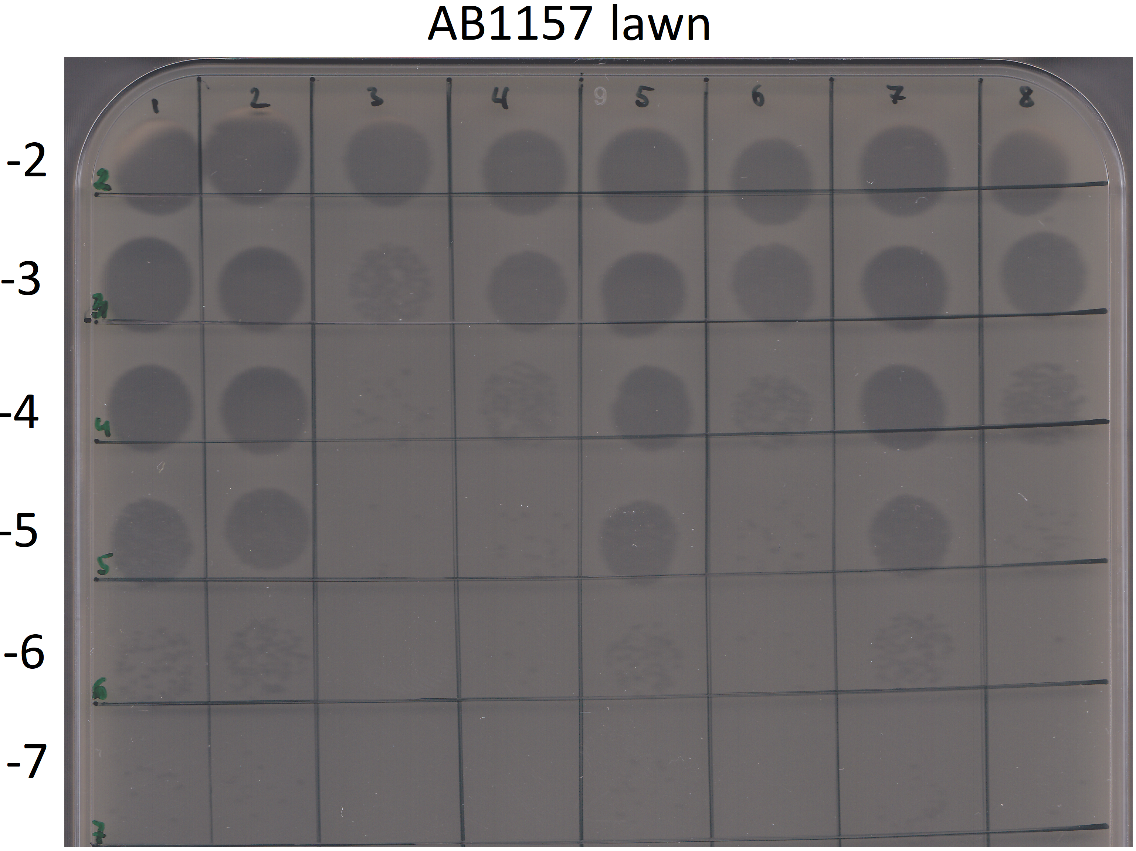


Supplementary figure 12. Effect of Ocr wt expression level on the methylations status of λ phage. Phage was obtained from lysogenic BREX- or BREX+ strains that overproduced wild-type Ocr at different concentrations of L-arabinose inducer. Phage lysates were tittered on BREX- and BREX+ cell lawns. The level of protection is shown as the ratio of phage titers obtained on non-restrictive (BREX-) relative to restrictive (BREX+) host. Decreased BREX protection against phage λ obtained in conditions with low levels of Ocr expression suggests the presence of BREX-specific modification. Inages of plates used to determine phage titers are presented in Supplementary Figure 13.

| BREX | - | + | + | + | + | + | + |
| --- | --- | --- | --- | --- | --- | --- | --- |
| L-arabinose | 67 mM | 67 mM | 6.7 mM | 666 μM | 67 μM | 6.7 μM | n/i |

Supplementary figure 13. Plating of λ phage induced from the BREX- or BREX+ cells carrying pBAD Ocr wt in the presence of different concentrations of L-arabinose inducer. Ten-fold dilutions of λ lysates are indicated on the left. Conditions of induction are indicated at the bottom. Representative plates from an experiment performed in triplicate are shown.


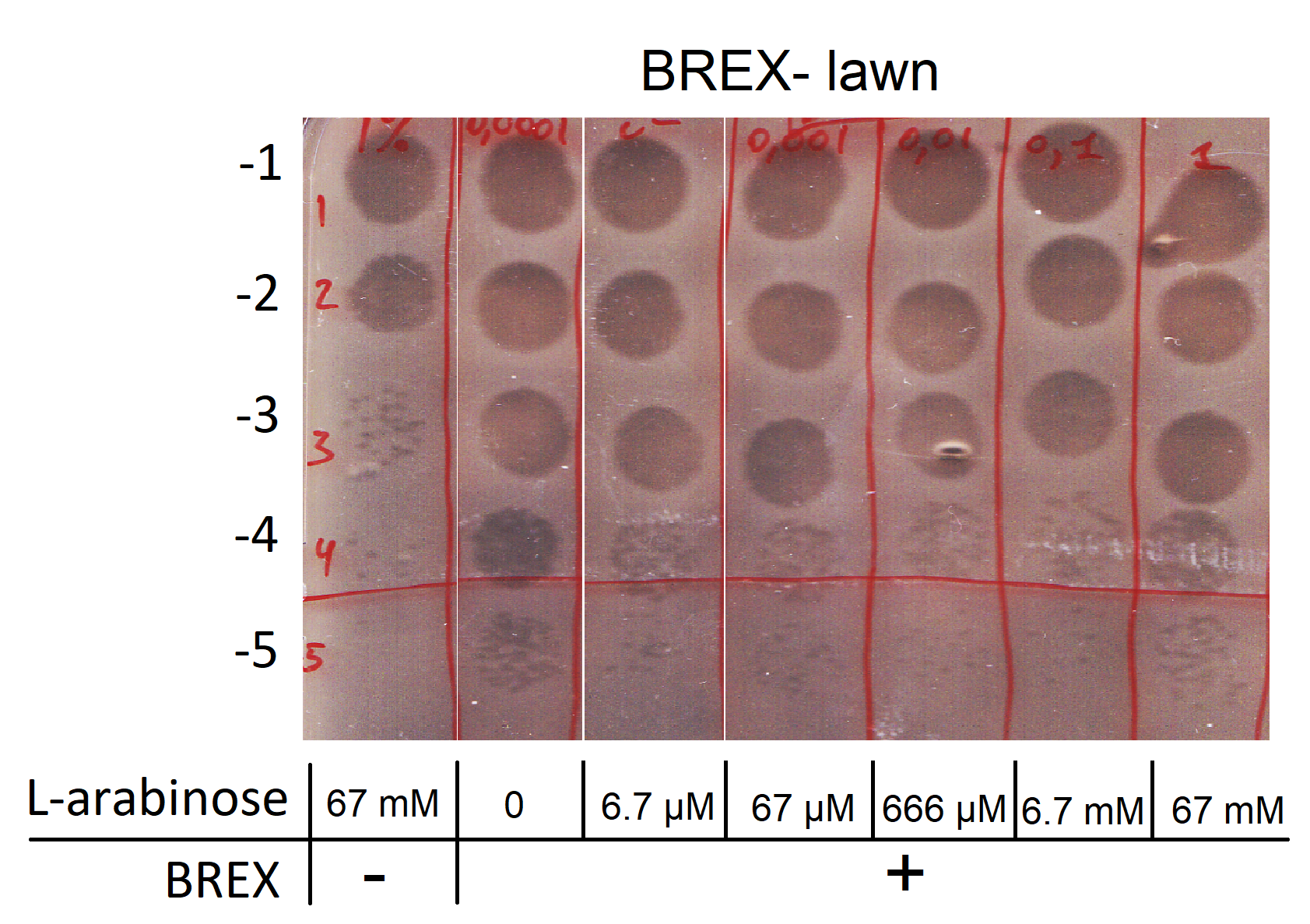

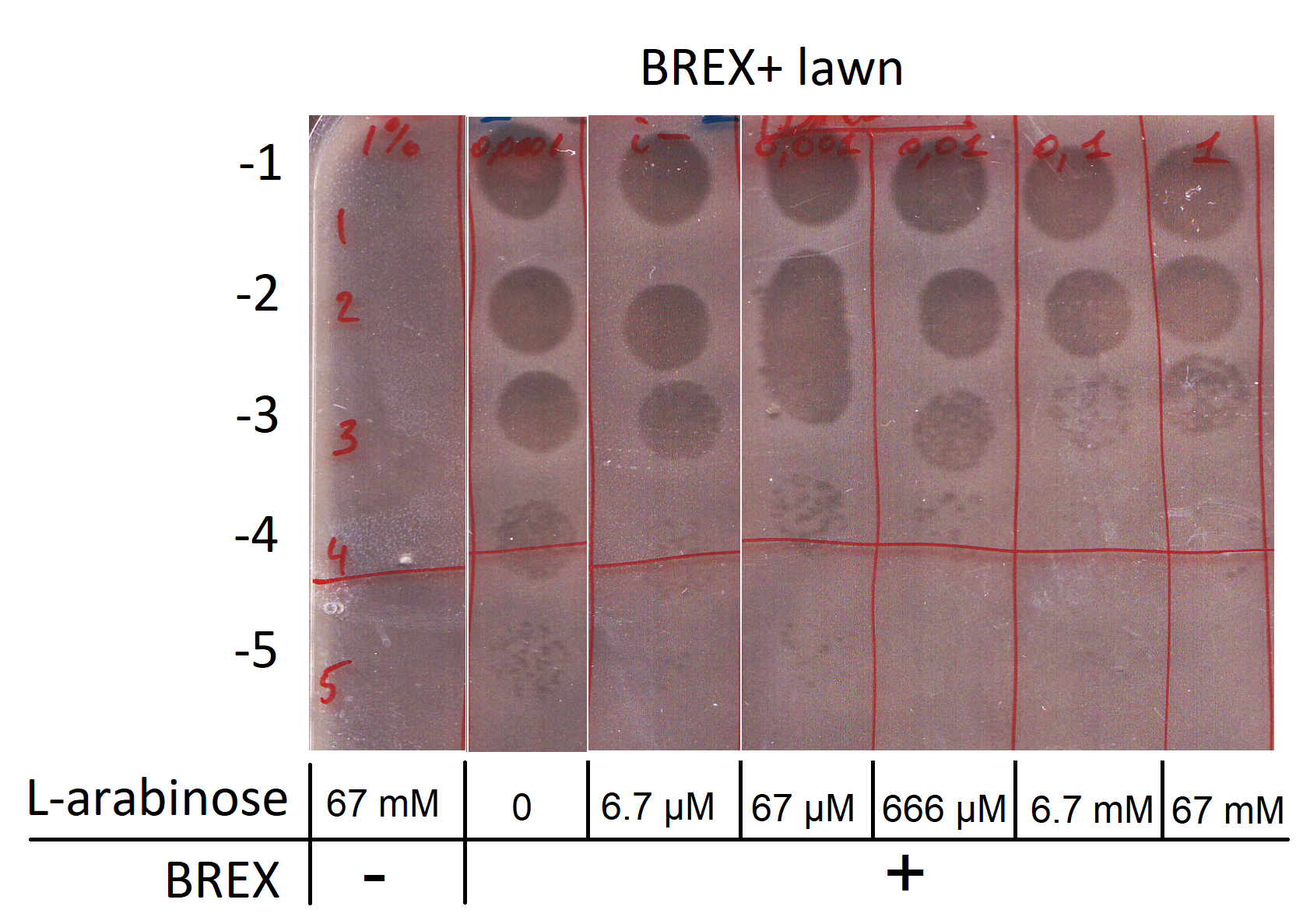

Supplement: gkaa290_Supplemental_Files [file gkaa290_supplemental_files.zip › Revised_Supplementary data_unmarked.docx]
